# Supplementary material for: Hierarchy and control of ageing-related methylation networks
Source: PLoS Comput Biol. 2021 Sep 17;17(9):e1009327. doi: 10.1371/journal.pcbi.1009327 (PMC8480875; doi:10.1371/journal.pcbi.1009327)
Supplement: S1 Text — Figure A: “Expected changes in the estimated age” Figure B: “Hierarchy of the methylation network based on the Skin-Blood clock” Figure C: “Hierarchy of the methylation network based on the Hannum’s clock” Figure D: “Control centrality and reach in the network defined based on the Skin-Blood clock” Figure E: “Control centrality and reach in the network defined based on the Hannum’s clock” Figure F: Scatter plot of expected change in the estimated age as a function of the the m-reach and the control centrality for the network based on the Skin-Blood clock. Figure G: Scatter plot of expected change in the estimated age as a function of the the m-reach and the control centrality for the network based on the Hannum’s clock. Figure H: Hierarchy of the methylation networks based on randomly chosen CpGs. Figure I: Control centrality and reach in methylation networks based on randomly chosen CpGs. Figure J: Hierarchy of the methylation networks based on the data set studied by Lehne et. al. Figure K: Control centrality and reach in methylation networks based on the data set studied by Lehne et. al. Figure L: Scatter plot of the expected change in the estimated age as a function of the the m-reach and the control centrality for the network based on the data studied by Lehne et. al. Figure M: Correlation between the m-reach obtained in the networks based on the Hannum et. al and on the Lehne et al. dataset. Figure N: Distribution of the top 10% of the CpGs according to the Lehne et al. hierarchy in the hierarchy based on the Hannum et al. network. Figure O: Distribution of the top 10% of the CpGs according to the Hannum et al. hierarchy in the hierarchy based on the Lehne et al. network. Figure P: Age distribution of the patients in the studied data sets. (PDF) [file pcbi.1009327.s001.pdf]

Hierarchy and control of ageing-related methylation networks: Supporting Information

Gergely Palla[1,2], Péter Pollner[1,2,*], Judit Börcsök[3,4], András Major[3], Béla Molnár[5], István Csabai[3]

1 MTA-ELTE Statistical and Biological Physics Research Group, Dept. of Biological Physics, Eötvös University, Budapest, Hungary

2 Health Services Management Training Centre, Semmelweis University, Budapest, Hungary.

3 Dept. of Physics of Complex Systems, ELTE Eötvös University, Budapest, Hungary

4 Danish Cancer Society Research Center, Copenhagen, Denmark

5 Molecular Medicine Research Group, Hungarian Academy of Sciences, Budapest, Hungary

* pollner@angel.elte.hu

S1 Distribution of the expected change in the estimated age

We have calculated the age derivative $\frac{\mathrm{d}a}{\mathrm{d}m_i}$ using Eq.(4) in the main text for each CpG in Horvath's clock, and have evaluated the corresponding change in the estimated age $|\Delta a|$ based on Eq.(5) in the main text. In Fig.A. we show the probability density $|\Delta a|$ obtained according to this framework at $\ell_{\max} = 0$ (purple) and at $\ell_{\max} = 4$ (green). Although the two distributions look quite similar, the distribution for $\ell_{\max} = 4$ seems to have more mass in the $|\Delta a| > 3$ region, and also the outlier values are further apart from the rest of the distribution compared to the $\ell_{\max} = 0$ case. For the peaks in the distribution at the largest observed $|\Delta a|$ values we also indicate the corresponding CpGs with arrows. Interestingly, for the CpG with the largest $|\Delta a|$ at $\ell = 0$,

corresponding to *AGBL5*, the increase in ℓ is having only a minor effect on the calculated $\left|\frac{\mathrm{d}a}{\mathrm{d}m}\right|$ and $|\Delta a|$ values. However, as we can see from the figure *AGBL5* is surpassed by *SCGN*, *BAZ2A* and *UCKL1* at $\ell = 4$, and for these CpGs there is a large difference between the $|\Delta a|$ value at $\ell = 0$ and at $\ell = 4$.

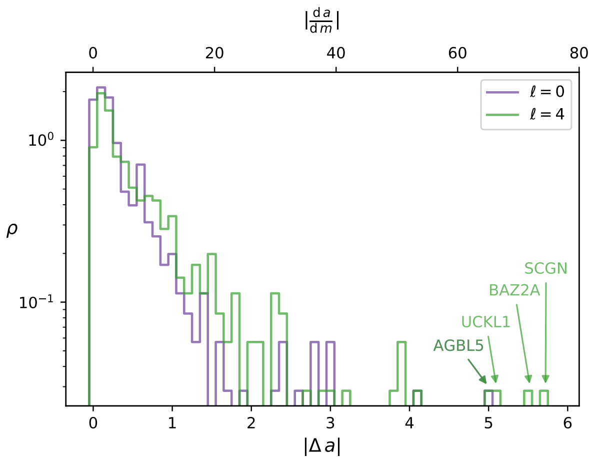

Fig A. Expected changes in the estimated age. The probability density ρ for the magnitude of the change in the estimated age, $|\Delta a|$, obtained from Eq. (5). The purple line shows the results when considering 'isolated' nodes, without any interaction between the CpGs, whereas the green line is obtained when taking into account all paths between the nodes up to length $\ell_{\max} = 4$. Since Δa and the derivative of the estimated age, $\frac{\mathrm{d}a}{\mathrm{d}m}$ are simply proportional to each other in our framework, the top axis is showing the corresponding value of $\left|\frac{\mathrm{d}a}{\mathrm{d}m}\right|$.

S2 Hierarchy and control properties of the methylation networks based on further epigenetic clocks

Although Horvath's clock is probably the most widely known epigenetic age estimator [1, 2],, there were further alternative epigenetic clocks proposed in the literature. Here we examine the hierarchical and control properties of the methylation networks corresponding to two prominent examples, given by the Skin-Blood clock [3] and Hannum's clock [4]. We made the sets of CpGs included in these clocks subject to the same analysis pipeline as detailed in the main text, that can be summarised as follows:

- Applying Lasso-CV to the methylation data narrowed down to the set of CpGs

taking part in the given epignetic clock, where the non-zero regression coefficients define the weighted links of the network.

- Searching for the optimal link weight threshold based on the concept of efficiency, and applying a weight threshold where the efficiency is maximal in order to make the network more sparse.
- Calculating the GRC hierarchy measure and comparing the result to the GRC in link randomised counterparts of the network.
- Evaluation of the control centrality of the nodes at different link weight thresholds and examining the correlation between the control centrality and m-reach.
- Calculation of the age derivative for the methylation levels based on the regression coefficients using equations analogous to eq.(4) in the main text, and evaluating the change in the expected age when the methylation level is changed.

In Fig.B we show the GRC of the Skin-Blood clock network (consisting of $N = 391$ nodes), together with the GRC distribution in the configuration network ensemble with the same degree distribution for m parameters between $m = 2$ and $m = 5$. The results indicate that this network is strongly hierarchical, in a fashion similar to the network between the CpGs of Horvath's clock. In Fig.C we display the analogous results for Hannum's clock (consisting of $N = 71$ CpGs). Although here the GRC for the original network is not far from the mean of the randomised networks at $m = 2$, for larger m values the difference becomes very large on the scale of the standard deviation of the randomised ensemble, similarly to the previous epigenetic clocks. According to that, the methylation network defined by Hannum's clock is also strongly hierarchical.

In Figs.D-E. we plot the m-reach at $m = 3$ as a function of the control centrality averaged over methylation networks obtained at different link weight thresholds for the Skin-Blood clock and Hannum's clock, respectively. Both figures display a clear positive correlation between the position in the hierarchy (defined by the m-reach) and the control centrality, similarly to the behaviour observed for the network corresponding to Horvath's clock, studied in the main text.

Finally, in Figs.F-G. we show the scatter plot of the magnitude of the change in the estimated age $|\delta a|$ calculated in the framework governed by Eqs.(4-5) in the main text,

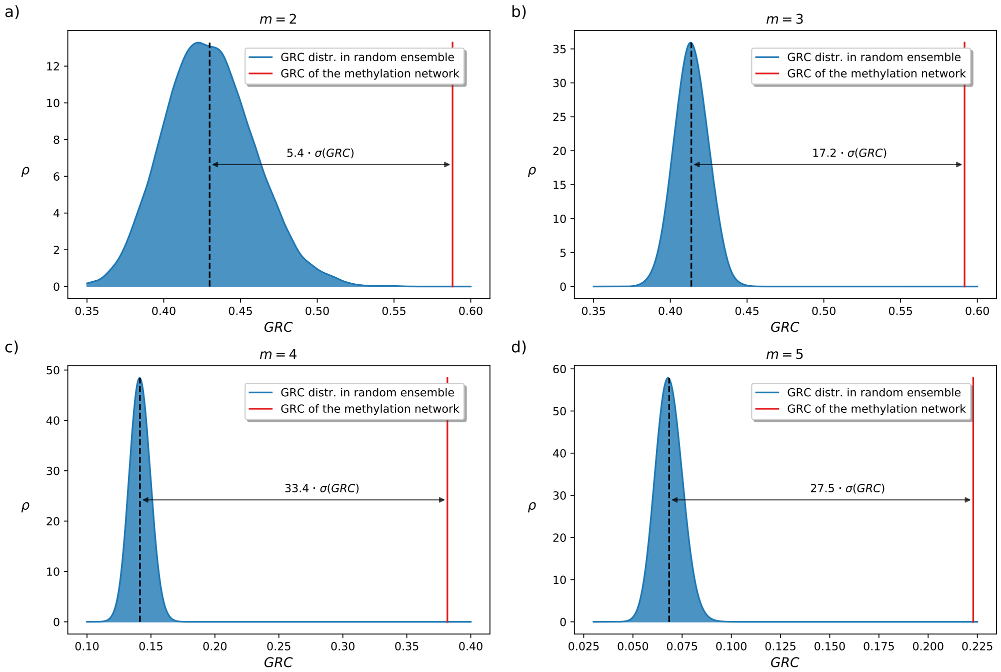

Fig B. Hierarchy of the methylation network based on the Skin-Blood clock. We show the GRC(m) measured for the network (red) together with probability density ρ(GRC) of the corresponding values in a link randomised ensemble of 10,000 networks (blue) at $m = 2$ (panel a), $m = 3$ (panel b), $m = 4$ (panel c), and $m = 5$ (panel d).

as a function of the m-reach and the control centrality of the node at which the starting methylation level perturbation is initiated. These figures show a great deal of similarity with Fig.7. in the main text obtained for Horvath's clock, and indicate that perturbing the methylation level nodes with higher m-reach and/or larger control centrality has a higher chance for achieving a pronounced shift in the estimated age in these networks as well.

S3 Hierarchy and control properties of methylation networks between randomly chosen CpGs

In order to extend our studies even further, we have investigated the hierarchical and control properties of methylation networks consisting of randomly chosen CpGs as well. During this analysis for each sample we have selected 353 CpGs from the 450k methylation array uniformly at random, and applied the usual framework for extracting the methylation network between them. After calculating the GRC for these networks,

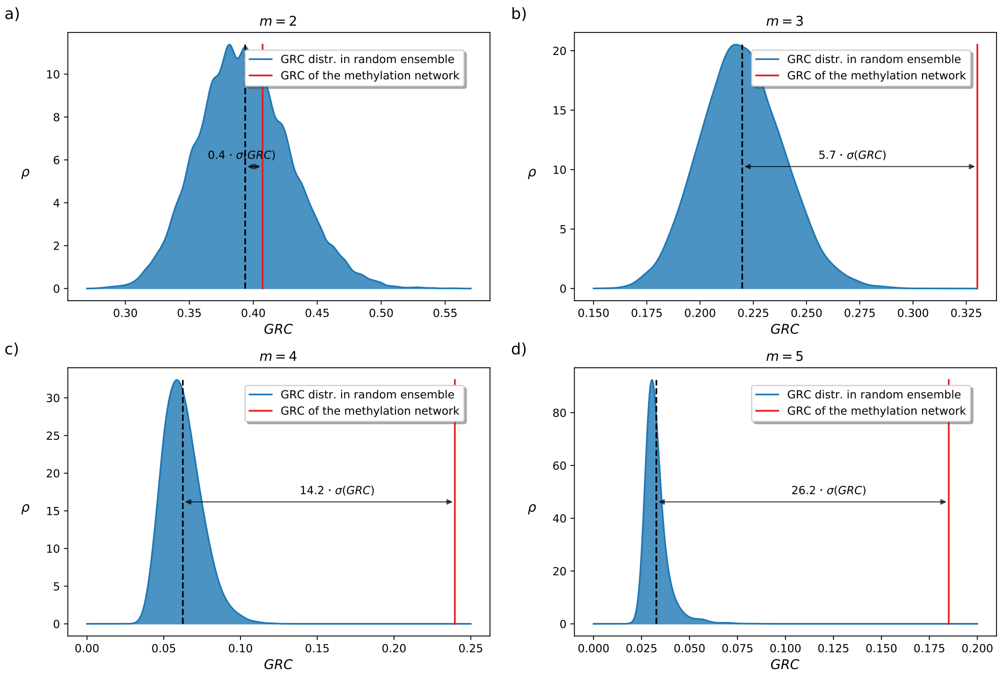

Fig C. Hierarchy of the methylation network based on Hannum's clock. We show the GRC(m) measured for the network (red) together with probability density ρ(GRC) of the corresponding values in a link randomised ensemble of 20,000 networks (blue) at $m = 2$ (panel a), $m = 3$ (panel b), $m = 4$ (panel c), and $m = 5$ (panel d).

we applied the same link-randomisation procedure as in case of the previously studied networks corresponding to epigenetic clocks, in order to obtain a base-line ensemble of graphs with no additional structure beyond the degree distribution.

In Fig.H. we compare the GRC distribution of the original methylation networks (shown in orange) with the same distribution calculated for the ensemble of their link-randomised counterparts (displayed in blue). We examined altogether 150 random methylation networks, and for each of them, the link-randomisation procedure was applied 50 times, yielding 7500 samples for the base-line distribution. According to Fig.H., there is a clear separation between the two distributions, where the hierarchy measure for the link-randomised networks is on average lower compared to the original methylation networks. In this figure we also re-plotted the GRC results for Horvath's clock from Fig.2. in the main text (shown in green). Based on this we can see that the methylation network of Horvath's clock has hierarchy values above the average compared to random methylation networks at all m values, however in the mean time it is also clear that its GRC value is not an outlier from the perspective of the overall

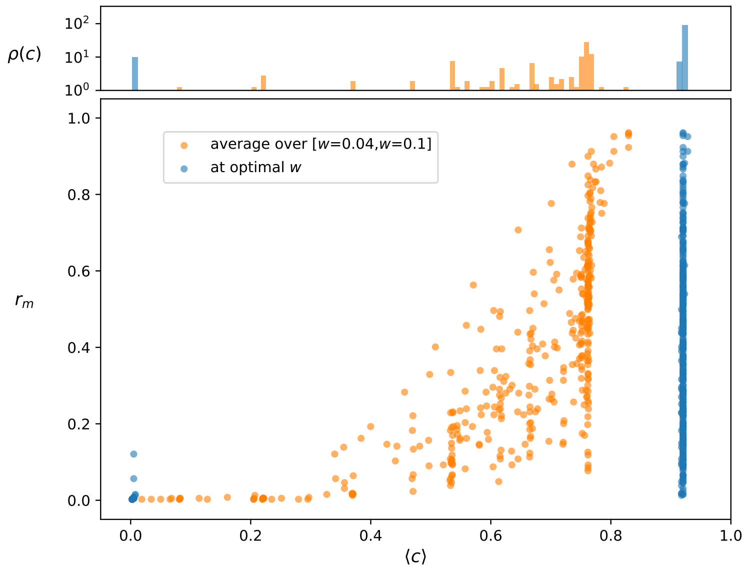

Fig D. Control centrality and reach in the network defined based on the Skin-Blood clock. The main panel shows the reaching centrality r_m at $m = 3$ as a function of the relative control centrality c. Each symbol in the plot is corresponding to an individual CpG dinucleotide (node in the methylation network). In blue we show the results for the methylation network at the optimal weight threshold w^*, whereas in case of the orange symbols $C(i)$ was averaged for the individual nodes over 50 different networks obtained by changing the w^* parameter in the $[w^* = 0.04, w^* = 0.1]$ interval. The Pearson correlation coefficient between $\langle c \rangle$ and r_m is 0.46 for the optimal network, and 0.65 in case of the averaging scenario. The top panel displays the density of the normalised control centrality c for the two cases.

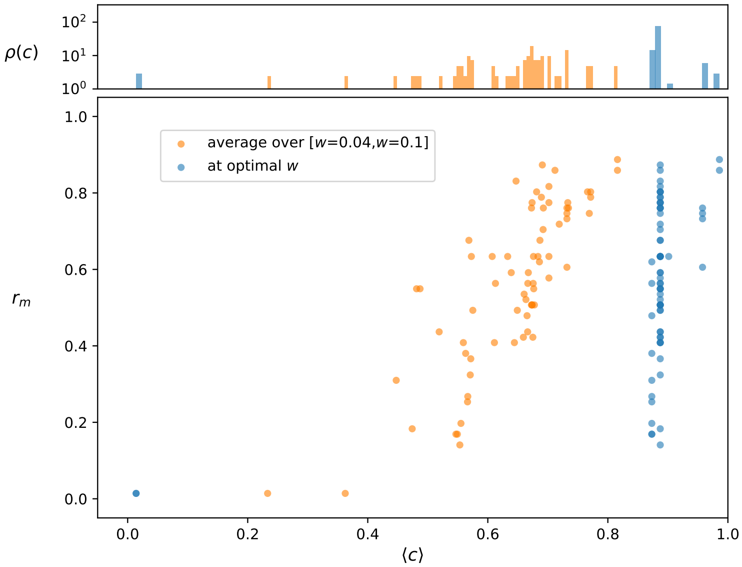

Fig E. Control centrality and reach in the network defined based on Hannum's clock. The main panel shows the reaching centrality r_m at $m = 3$ as a function of the relative control centrality c. Each symbol in the plot is corresponding to an individual CpG dinucleotide (node in the methylation network). In blue we show the results for the methylation network at the optimal weight threshold w^*, whereas in case of the orange symbols $C(i)$ was averaged for the individual nodes over 50 different networks obtained by changing the w^* parameter in the $[w^* = 0.04, w^* = 0.1]$ interval. The Pearson correlation coefficient between $\langle c \rangle$ and r_m is 0.49 for the optimal network, and 0.80 in case of the averaging scenario. The top panel displays the density of the normalised control centrality c for the two cases.

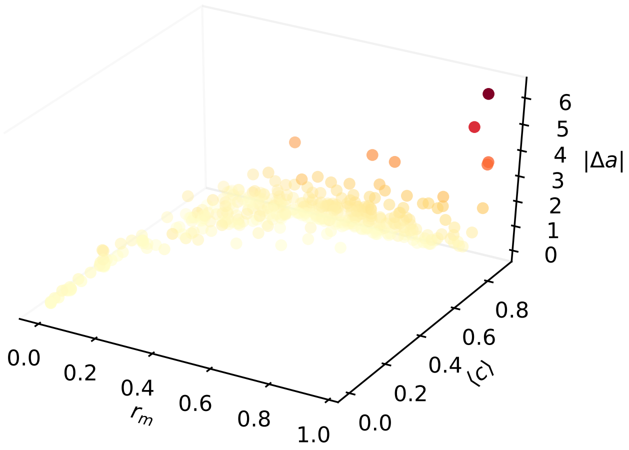

Fig F. Scatter plot of expected change in the estimated age as a function of the the m-reach and the control centrality for the network based on the Skin-Blood clock. The vertical axis is corresponding to $|\Delta a|$, calculated according to Eq.(5), where the initial perturbation Δm_i on the methylation level is equal to $2\,\langle\sigma(m)\rangle$ for all i, and the age derivative for node i is obtained from Eq.(4) at $\ell_{\max} = 4$. On the x axis we display the m-reach r_m at $m = 3$, whereas the y axis is corresponding to the average control centrality $\langle c\rangle$. The colouring of the symbols follows their vertical coordinate, with bright colours corresponding to low $|\Delta a|$ values, and darker shades representing a larger expected change in a.

GRC distribution of random methylation networks.

In Fig.I. we examine the control properties of the random methylation networks along the same line as we did for the epigenetic clock networks. The scatter plot of the m-reach as a function of $\langle c\rangle$ is indicating a positive correlation between the control centrality of the nodes and their position in the hierarchy, in a similar fashion to the control centrality results obtained for the previously studied networks.

S4 Methylation networks based on the Lehne et. al dataset

An additional possibility for generalising our results is to apply the analysis pipeline to further data-sets beside the methylation data studied in Ref. [4] by Hannum et. al, serving as the input for the studies outlined in the main paper. Along this line, in the present Section we switch to the data analysed by Lehne et. al in Ref. [5], listing altogether 2675 individuals (36 samples measured in duplicate) aged from 23 to 75, with a median age of 50, including 871 female and 1840 male participants. The methylated

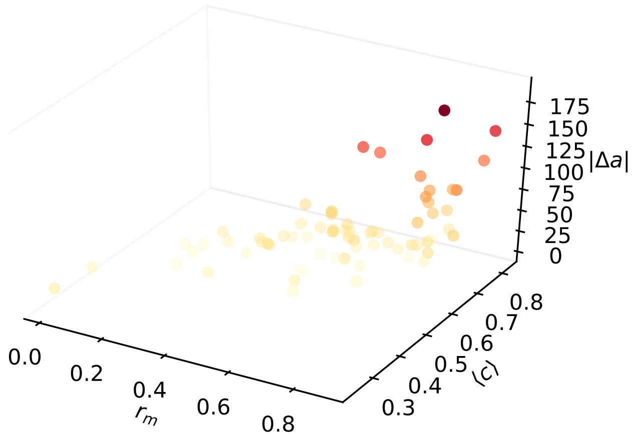

Fig G. Scatter plot of expected change in the estimated age as a function of the the m-reach and the control centrality for the network based on Hannum's clock. The vertical axis is corresponding to $|\Delta a|$, calculated according to Eq.(5), where the initial perturbation Δm_i on the methylation level is equal to $2\langle\sigma(m)\rangle$ for all i, and the age derivative for node i is obtained from Eq.(4) at $\ell_{\max} = 4$. On the x axis we display the m-reach r_m at $m = 3$, whereas the y axis is corresponding to the average control centrality $\langle c \rangle$. The colouring of the symbols follows their vertical coordinate, with bright colours corresponding to low $|\Delta a|$ values, and darker shades representing a larger expected change in a.

and unmethylated signal intensity values were downloaded from the NCBI's Gene Expression Omnibus (GEO) using the "GSE55763" accession number. The minfi (v1.32.0) R package Ref. [6] was used to calculate β values from the raw intensities with the *MethylSet()* and *ratioConvert()* functions. Technical replicates (36 samples measured in duplicate) and samples in which any of the Horvath CpGs (n=11) had a missing value and were excluded.

The extraction of the methylation network followed the same steps as in case of all other examined methylation input data. In Fig.J. we show the GRC(m) measured in the obtained network, compared to the distribution in link-randomised networks with the same degree sequence at various m parameters. According to the results, the GRC in the original network is outstandingly larger compared to the average of the randomised ensemble, analogously to the behaviour of the GRC in the previously examined networks.

In Fig.K. we show the relation between the control centrality and the m-reach in a similar fashion as for the previously examined networks, displaying the results both at the optimal weight threshold (blue) and in a wider range of link weight thresholds

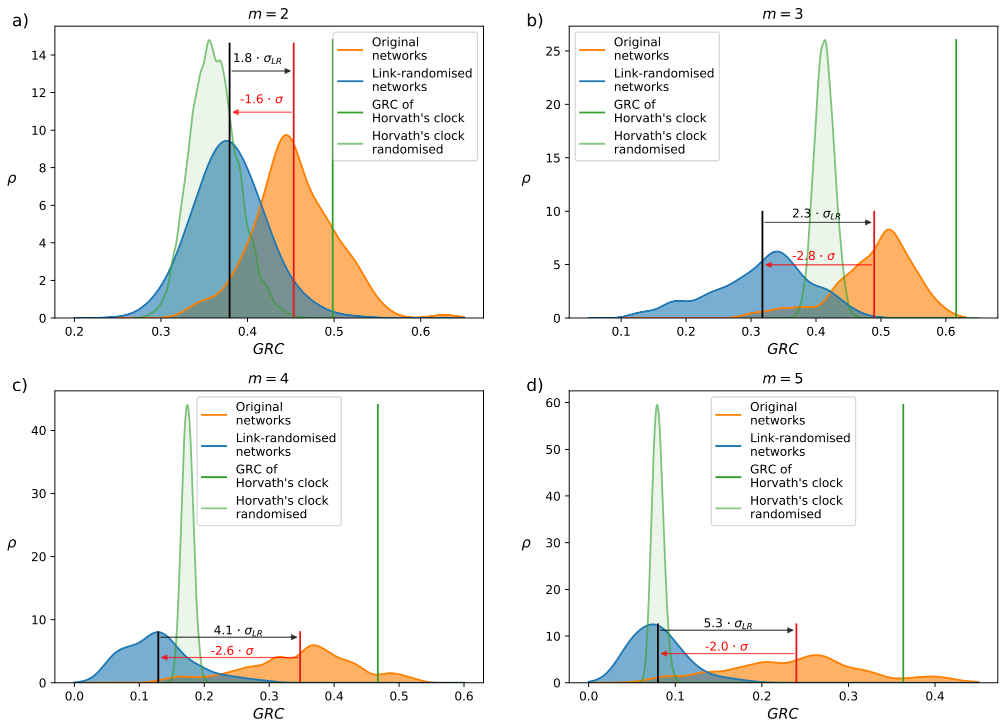

Fig H. Hierarchy of the methylation networks based on randomly chosen CpGs. We show the GRC density distribution for 150 networks in orange, where each sample was consisting of 353 CpGs chosen uniformly at random from the 450k array. The ρ(GRC) corresponding to the link-randomised counterparts of these networks is displayed in blue (based on 7500 instances). For comparison, we also re-plot the results obtained for the network based on Horvath's clock in green (Fig. in the main text). The panels list the results obtained at different m parameters, with $m = 2$ in panel a), $m = 3$ in panel b), $m = 4$ in panel c), and $m = 5$ in panel d).

(orange). The overall pattern of the point clouds is quite similar to the results obtained in the previously studied systems, indicating a positive correlation between the control centrality of the nodes and their position in the hierarchy.

We also examined the effect of the perturbation of the methylation levels on the predicted age according to Horvath's clock, following the same framework as in the main text for the network based on the data examined in the study by Hannum et. al. The expected change in the predicted age, $|\Delta a|$ was calculated according to Eq.(5) in the main text, where the initial perturbation Δm_i on the methylation level was set to $2\,\langle\sigma(m)\rangle$ for all i. The age derivative for node i was obtained according to Eq.(4) in the main text at $\ell_{\max} = 4$. The scatter plot for $|\Delta a|$ as a function of r_m and $\langle c\rangle$ is provided in Fig.L, showing a similar pattern compared to the analogous plots for the previously examined methylation networks. According to that, notable changes in the estimated

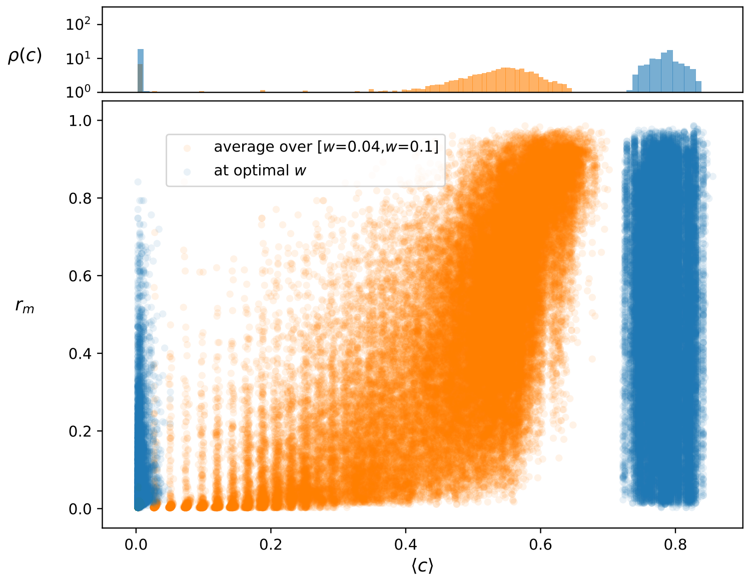

Fig I. Control centrality and reach in methylation networks based on randomly chosen CpGs. The main panel shows the reaching centrality r_m at $m = 3$ as a function of the relative control centrality c for the methylation networks studied in Fig.H. Each symbol in the plot is corresponding to an individual CpG dinucleotide. In blue we show the results for the methylation networks at their optimal weight threshold w^*, whereas in case of the orange symbols $C(i)$ was averaged for the individual nodes over 50 different networks obtained by changing the w^* parameter in the $[w^* = 0.04, w^* = 0.1]$ interval. The Pearson correlation coefficient between $\langle c \rangle$ and r_m is 0.50 for the optimal network, and 0.75 in case of the averaging scenario. The top panel displays the density of the normalised control centrality c for the two cases.

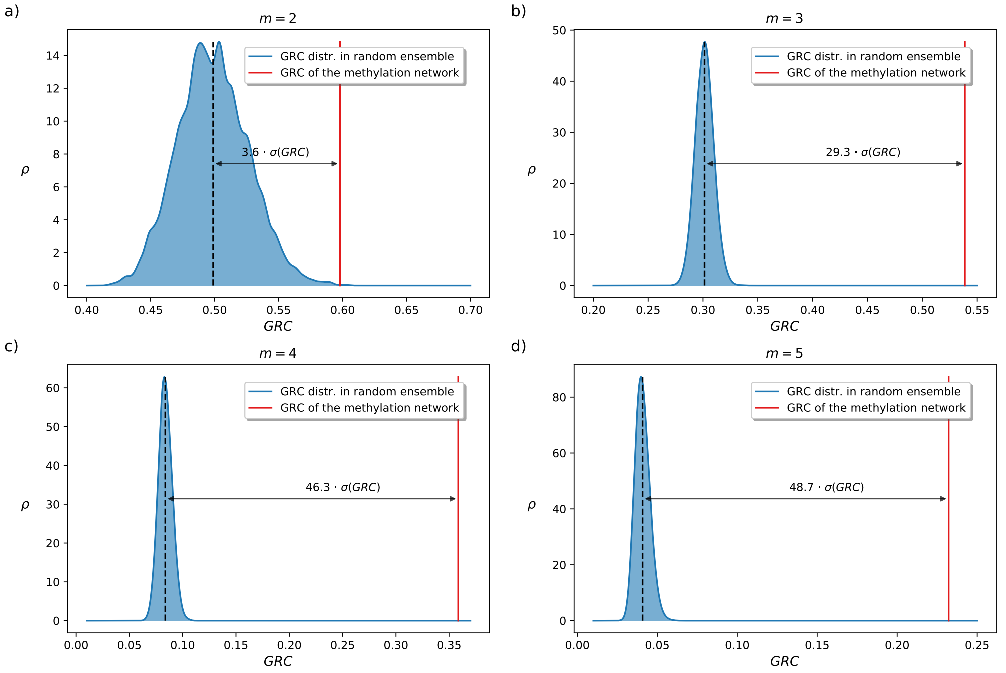

Fig J. Hierarchy of the methylation networks based on the data set studied by Lehne et. al. The GRC(m) measured for the network (red) is displayed together with the probability density ρ(GRC) of the corresponding values in a link randomised ensemble of 5,000 networks (blue) at $m = 2$ (panel a), $m = 3$ (panel b), $m = 4$ (panel c), and $m = 5$ (panel d).

age are likely to occur when perturbing the methylation level of nodes with larger m-reach and/or higher control centrality in this system as well, similarly to the results seen for the previously studied networks.

A remaining question of interest is whether the hierarchy obtained for the Lehne et. al data is providing a similar ordering between the nodes as in case of the hierarchy for the Hannum et. al data studied in the main paper? Since the position in the hierarchy is based on the m-reach, we can study this question by examining the correlation between the m-reach in the two networks. Along this line, in Fig.M. we show the scatter plot of the m-reach in the network based on the data studied by Lehne et. al, as a function of the m-reach in the network based on the data from Hannum et. al (studied in the main text). In this figure each dot is corresponding to one of the 353 CpGs appearing in Horvath's clock, and according to the kernel density contours (indicated by the coloured background) the measured m-reach at $m = 3$ in the two separate networks display a notable positive correlation, with a correlation coefficient equal to $C = 0.75$.

Another possibility for comparing the two hierarchies is to extract the top nodes

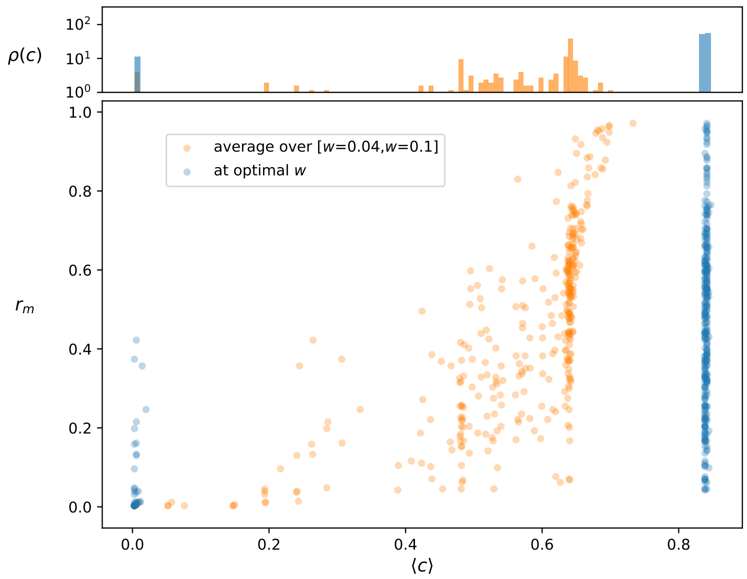

Fig K. Control centrality and reach in methylation networks based on the data set studied by Lehne et. al. The main panel shows the reaching centrality r_m at $m = 3$ as a function of the relative control centrality c. Each symbol in the plot is corresponding to an individual CpG dinucleotide (node in the methylation network). In blue we show the results for the methylation network at the optimal weight threshold, whereas in case of the orange symbols C(i) was averaged for the individual nodes over 60 different networks obtained by changing the w^* parameter in the $[w^* = 0.04, w^* = 0.1]$ interval. The Pearson correlation coefficient between $\langle c \rangle$ and r_m is 0.51 for the optimal network, and 0.71 in case of the averaging scenario. The top panel displays the density of the normalised control centrality c for the two cases.

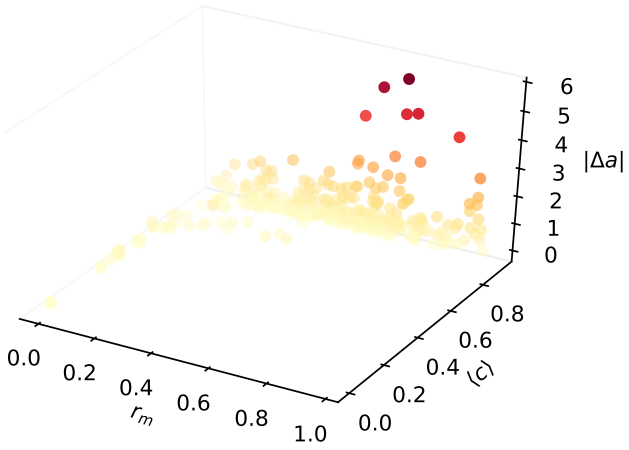

Fig L. Scatter plot of the expected change in the estimated age as a function of the the m-reach and the control centrality for the network based on the data studied by Lehne et. al. The vertical axis is corresponding to $|\Delta a|$ calculated at $\ell_{\max} = 4$, the x axis is showing the m-reach r_m at $m = 3$, an the y axis is displaying the average control centrality $\langle c \rangle$. The colouring of the symbols follows their vertical coordinate, with bright colours corresponding to low $|\Delta a|$ values, and darker shades representing a larger expected change in a.

according to one, and examine their distribution in the other, in a somewhat analogous way as in case of the mixed hierarchies studied in Fig.8. in the main paper. The rationale behind this approach is that since the most influential nodes in a hierarchy are at the top, when judging the extent of similarity between two hierarchies, a very important aspect is whether the position of these top nodes falls close within the two systems or not? Along this line, in Fig.N. we show the distribution of the top 10% of the nodes according to the Lehne et al. hierarchy in the hierarchy based on the Hannum et al. network, whereas in Fig.O. we display the analogous distribution arising in the hierarchy based on the Lehne et al. network, when locating the top nodes according to the Hannum et al. hierarchy. In both cases, the distribution is narrowly peaked close to the top of the given hierarchy, thus, the two hierarchies show a reasonably good match with each other according to this approach as well.

Finally, we note that a quite plausible reason for the slight difference between the two hierarchies observed according to Figs.M-O. is the large discrepancy between the age distribution of the patients in the two cohorts, as indicated by Fig.P. As shown by the blue curve, in case of the data set studied by Hannum et al., the age of the patients is spanning between 19 and 101 years, and quite a large fraction of the patients had an

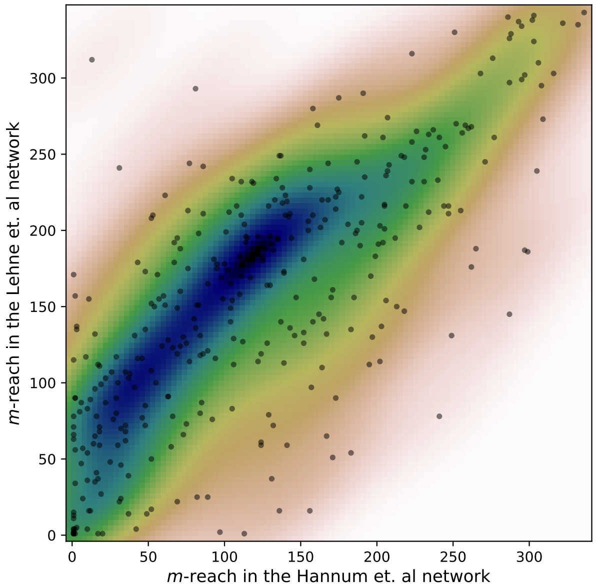

Fig M. Correlation between the m-reach obtained in the networks based on the Hannum et. al and on the Lehne et al. dataset. We show the scatter plot of the m-reach at $m = 3$ for the nodes in the Lehne et al. network as a function of the m-reach obtained in the Hannum et al. network. The Pearson correlation coefficient is $C = 0.75$.

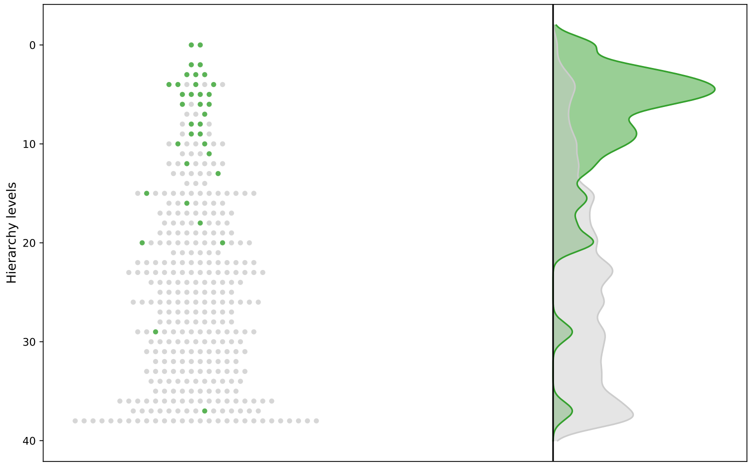

Fig N. Distribution of the top 10% of the CpGs according to the Lehne et al. hierarchy in the hierarchy based on the Hannum et al. network. The top nodes according to the Lehne et al. hierarchy are colored green, and the density distributions of these nodes and all nodes (colored grey) are shown on the right.

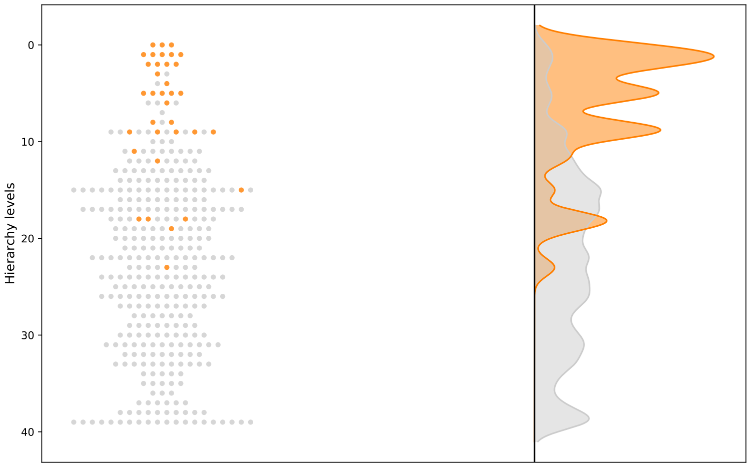

Fig O. Distribution of the top 10% of the CpGs according to the Hannum et al. hierarchy in the hierarchy based on the Lehne et al. network. Similarly to Fig.N., the orange color marks the top nodes according to the Hannum et al. hierarchy, and the corresponding densities are displayed on the right.

age above 75 years old. In contrast, for the data set studied by Lehne et al. (orange curve), the age of the patients can fall only between 23 and 75 years old. Since the methylatiton profile is changing with ageing, both the methylation network and the hierarchy constructed from the methylation network is expected to show differences for the two data sets, due to the relatively large fraction of elderly people present in one that is completely missing in the other. In this light, the relatively large correlation between the m-reach for the two networks shown in Fig.M and the convincing match between the top parts of the hierarchies observed in Figs.N-O. indicate that our framework for the study of the methylation network between CpGs is robust in general.

References

1. Horvath S. DNA methylation age of human tissues and cell types. Genome Biol. 2013;14:R115. doi:https://dx.doi.org/10.1186/gb-2013-14-10-r115.

2. Horvath S, Raj K. DNA methylation-based biomarkers and the epigenetic clock theory of ageing. Nature Reviews Genetics. 2018;19:371–384.

3. Horvath S, Oshima J, Martin GM, Lu AT, Quach A, Cohen H, et al. Epigenetic clock for skin and blood cells applied to Hutchinson Gilford Progeria Syndrome and ex vivo studies. Aging. 2018;10:1758–1775. doi:10.18632/aging.101508.

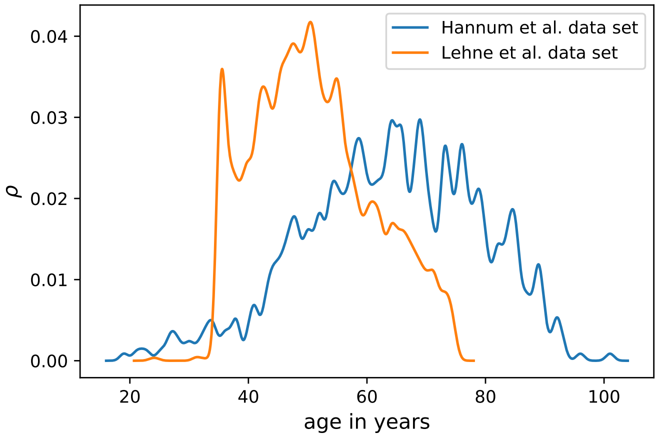

Fig P. Age distribution of the patients in the studied data sets. We plot the density distribution ρ of patients according to their age (measured in years) for the methylation data set studied by Hannum et. al in Ref. [4] (blue curve) together with the analogous distribution for the methylation data set analysed by Lehne et. al in Ref. [5] (orange curve).

4. Hannum G, Guinney J, Zhao L, Zhang L, Hughes G, Sadda S, et al. Genome-wide Methylation Profiles Reveal Quantitative Views of Human Aging Rates. Molecular Cell. 2013;49(2):359 – 367. doi:https://doi.org/10.1016/j.molcel.2012.10.016.

5. Lehne B, Drong AW, Loh M, Zhang W, Scott WR, Tan ST, et al. A coherent approach for analysis of the Illumina HumanMethylation450 BeadChip improves data quality and performance in epigenome-wide association studies. Genome Biol. 2015;16:37. doi:10.1186/s13059-015-0600-x.

6. Aryee MJ, Jaffe AE, Corrada-Bravo H, Ladd-Acosta C, Feinberg AP, Hansen KD, et al. Minfi: a flexible and comprehensive Bioconductor package for the analysis of Infinium DNA methylation microarrays. Bioinformatics. 2014;30(10):1363–1369. doi:10.1093/bioinformatics/btu049.
