# Supplementary material for: Hierarchy and control of ageing-related methylation networks
Source: PLoS Comput Biol. 2021 Sep 17;17(9):e1009327. doi: 10.1371/journal.pcbi.1009327 (PMC8480875; doi:10.1371/journal.pcbi.1009327)
Supplement: Attachment — Submitted filename: Palla et al.pdf [file pcbi.1009327.s002.pdf]

Palla *et al*. have produced a manuscript entitled "Hierarchy and control of ageing-related methylation networks." In this paper they have extracted an interaction network from the CpGs employed in the Horvath clock. Unsurprisingly, this shows some hierarchical organisation is present. They then go on to discuss how modifying the clock will led to age 'reversal'. Unfortunately, there are significant issues in the design and conclusions drawn from this study, due to imprecise understanding of the ageing biology of the epigenome, as well as the construction and interpretation of the Horvath clock. The researchers have performed a network analysis focused only the 353 CpGs from this specific clock, without acknowledging that these CpGs themselves are not uniquely special in regard to their functionality. The discussion of age-reversal gives these methylation sites a definitely active role in the ageing process which they do not possess. My concerns are listed below.

<u>Major</u>

1. The statements in the Abstract that it is "plausible to assume that by proper adjustment of these switches age may be tuned" and that "biological clock can be changed or even reversed" – are counter to the current understanding of the field and imply that the clock itself is driving ageing rather than a 'biomarker' of the ageing process and the plethora of ageing-related changes it is capturing[1]. The clock itself is used to measure the impact of potential interventions[2].
2. Furthermore, the statement that "adjustment of one leads to a cascade of changes at other sites" is not surprising if one understands what biological and connected epigenetic changes will be represented, as in this case of blood tissue derived DNA[3].
3. The statement in Abstract and elsewhere that 'we locate the most important CpGs' ignores the fact that they limit their analysis to only the 353 CpG from the total DNA methylome of 28 million CpGs to begin with. As Horvath has stated there is no evidence that the CpGs in the Horvath clock are especially functional over and above many other CpGs and reasonable clocks can be constructed from even a random selection of CpGs - there are abundant potential CpGs that can be exploited in clocks[3]. The statement "largest influence" and "which may also play a crucial role in the process of ageing" (Introduction, Line 94) again implies these small fraction of 353 CpGs are uniquely special[4].
4. Age-related change in DNA methylome is in fact widespread with up to 15 – 30% of all CpG sites in the genome associated with age-related changes and these are not all called 'clock CpGs' (Introduction line 18). Change can be random fashion due to epigenomic drift[5], directional, or show increased variability with age[6]. Also, the statement regarding the directionality of "clock CpGs that are hypermethylated" (Introduction line 35) is an oversimplification. Teschendorff *et al*. identified an enrichment in an early promoter-focused array for age-related CpGs that were hypermethylating in the Targets of Polycomb Target gene promoters, but genome-

wide hypomethylation predominates. Both hypo- and hypermethylated loci contribute to the various published clocks.

5. The statement in the Introduction that there are "connections between the CpGs themselves' (line 75) is as expected. Clearly all well-known ageing effects lead to co-ordinated changes across the entire DNA methylome – these include those driven by cell-type specific epigenomics where changes in cell proportion will led to variation (including the age-related myeloid skew[7], T cell exhaustion)[8], polycomb target hypermethylation[9], bivalent domain hypermethylation[10], *etc*. These known systemic effects will be seen as networks of age-related change.
6. Distinct biological processes drive the observed age-related hypermethylation and hypomethylation. Furthermore, the baseline DNA methylation state is strongly driven by genetics being highly CpG density dependent[11].
7. The statement (line 53) that "we cannot really point out any of these CpGs as being more important than others" is as completely expected in the way that the elastic net regression Horvath clock was designed. CpGs were selected not for their individual strength but chosen for their power to work collectively to parsimoniously capture ageing over the lifecourse. In fact, this is clearly demonstrated by the fact the strongest and most robust individual CpG pan-tissue changes from the *ELOVL2* locus[12,13] were not included in the clock. Additionally, an accurate clock has been devised using just 3 CpGs[14].
8. The discussion of "control properties" of CpGs is consistent with the Elastic Net picking those CpGs that work well together. Thus, the results regarding network identification and properties have ignored this and the limited CpGs this has been exacted from *e.g.* Results (line 112). Why were not all the ~850,000 CpGs from the EPIC array analysed in the network analysis rather than just 353? Conclusion statements regarding how a "network approach can bring new insight into methylation-related studies, providing a very interesting direction for further research" (Line 389) are clearly limited when restricted to only these 353 CpGs and known biology not taken into account.
9. The authors need to explain and understand more precisely what the concept of 'biological age' and predicators of this represent[15]. The initial Horvath clock was devised as an attempt at a 'pan-tissue' clock (which it was highly successful in although caveats remain[16,17]). It is in fact a 'composite' clock[3] capturing both forensic and biological age but neither perfectly. The authors need to understand and integrate the current knowledge and issues regarding DNA methylation clocks - as discussed recently by the epigenomics community[4].
10. The statements regarding "Modifying the predicted age by perturbing the methylation network' need to be put in the context that they are interpreting a 'biomarker' of biological ageing.

11. Unclear what "more aligned with the 'natural direction of ageing'." (Line 283) means biologically?
12. In the Discussion the statement 'Horvath's clock is showing non-trivial hierarchical and control properties' – how is this unexpected? Furthermore, how would that be different from a random selection of array-derived CpG probes?
13. The statements regarding the functional implications of individual CpGs in the Discussion need to be more clearly caveated[8].
14. In the Conclusion (line 374) the statement "substantially more hierarchical compared to a random Graph" does not take into consideration the biological nature of these data.

<u>Minor</u>

1. English needs correcting throughout manuscript
2. Abstract – Grammar - "…biomarkers of ageing"
3. "specific CpG pairs" line 20 – CpG 'dinucleotides' is usually stated as more precise
4. Spelling line 33 - DNA methylation
5. Gene names are by convention written in italics – e.g. *UCKL1* gene (line 314) etc.

1. Horvath, S. & Raj, K. DNA methylation-based biomarkers and the epigenetic clock theory of ageing. Nat Rev Genet 19, 371-384 (2018).
2. Fahy, G.M. et al. Reversal of epigenetic aging and immunosenescent trends in humans. Aging Cell 18, e13028 (2019).
3. Field, A.E. et al. DNA Methylation Clocks in Aging: Categories, Causes, and Consequences. Mol Cell 71, 882-895 (2018).
4. Bell, C.G. et al. DNA methylation Aging Clocks: Challenges & Recommendations. Genome Biology (2019).
5. Feil, R. & Fraga, M.F. Epigenetics and the environment: emerging patterns and implications. Nature reviews. Genetics 13, 97-109 (2011).
6. Slieker, R.C. et al. Age-related accrual of methylomic variability is linked to fundamental ageing mechanisms. Genome Biol 17, 191 (2016).
7. Rimmelé, P. et al. Aging-like Phenotype and Defective Lineage Specification in SIRT1-Deleted Hematopoietic Stem and Progenitor Cells. Stem Cell Reports 3, 44-59 (2014).
8. Lappalainen, T. & Greally, J.M. Associating cellular epigenetic models with human phenotypes. Nat Rev Genet 18, 441-451 (2017).
9. Teschendorff, A.E. et al. Age-dependent DNA methylation of genes that are suppressed in stem cells is a hallmark of cancer. Genome Res 20, 440-6 (2010).
10. Rakyan, V.K. et al. Human aging-associated DNA hypermethylation occurs preferentially at bivalent chromatin domains. Genome Res 20, 434-9 (2010).
11. Baubec, T. & Schübeler, D. Genomic patterns and context specific interpretation of DNA methylation. Current Opinion in Genetics & Development 25, 85-92 (2014).
12. Garagnani, P. et al. Methylation of ELOVL2 gene as a new epigenetic marker of age. Aging Cell 11, 1132-4 (2012).

13. Slieker, R.C., Relton, C.L., Gaunt, T.R., Slagboom, P.E. & Heijmans, B.T. Age-related DNA methylation changes are tissue-specific with ELOVL2 promoter methylation as exception. Epigenetics & Chromatin 11, 25 (2018).
14. Weidner, C.I. et al. Aging of blood can be tracked by DNA methylation changes at just three CpG sites. Genome Biol 15, R24 (2014).
15. Jylhävä, J., Pedersen, N.L. & Hägg, S. Biological Age Predictors. EBioMedicine 21, 29-36 (2017).
16. Horvath, S. et al. Epigenetic clock for skin and blood cells applied to Hutchinson Gilford Progeria Syndrome and ex vivo studies. Aging (Albany NY) 10, 1758-1775 (2018).
17. Zhang, Q. et al. Improved precision of epigenetic clock estimates across tissues and its implication for biological ageing. Genome Med 11, 54 (2019).
