# Supplementary material for: Hierarchy and control of ageing-related methylation networks
Source: PLoS Comput Biol. 2021 Sep 17;17(9):e1009327. doi: 10.1371/journal.pcbi.1009327 (PMC8480875; doi:10.1371/journal.pcbi.1009327)
Supplement: Attachment — Submitted filename: AnswersToReferees.pdf [file pcbi.1009327.s003.pdf]

Péter Pollner
Health Services Management Training Centre, Semmelweis University
H-1125, Kútvölgyi út 2,
Hungary

August 25, 2020

Editor
PLOS Computational Biology

Dear Editor of *PLOS Computational Biology*,

thank you very much for your e-mail message of the 18th of June 2020, including two extremely helpful referee reports on our manuscript PCOMPBIOL-D-20-00368 titled "Hierarchy and control of ageing-related methylation networks". The first Referee raised heavy but in the mean time absolutely fair criticism regarding certain aspects of our manuscript. The second Referee was slightly more positive, mentioning that our work is interesting, however, also had a couple of questions and remarks.

We have substantially rewritten the manuscript by taking into account the points raised by the Referees. We thank both Referees for their outstanding work and the very valuable comments, making the revised version of the paper significantly better, which we hope now is suitable for publication. Our point-by-point response to the reviewer comments (reproduced in italics) are given below.

Response to Reviewer 1:

We thank the Referee for the careful and detailed examination of the manuscript and the extremely valuable comments, which indeed have helped making our paper better. We are truly grateful for the 17 bibliographic references included in the report that we now also cited in the revised version of the paper. Our detailed answers to the points raised are the following:

Palla et al. have produced a manuscript entitled "Hierarchy and control of ageing-related methylation networks." In this paper they have extracted an interaction network from the CpGs employed in the Horvath clock. Unsurprisingly, this shows some hierarchical organisation is present. They then go on to discuss how modifying the clock will led to age 'reversal'. Unfortunately, there are significant issues in the design and conclusions drawn from this study, due to imprecise understanding of the ageing biology of the epigenome, as well as the construction and interpretation of the Horvath clock. The researchers have performed a network analysis focused only the 353 CpGs from this specific clock, without acknowledging that these CpGs themselves are not uniquely special in regard to their functionality. The discussion of age-reversal gives these methyla-

tion sites a definitely active role in the ageing process which they do not possess. My concerns are listed below.

Major

1. The statements in the Abstract that it is "plausible to assume that by proper adjustment of these switches age may be tuned" and that "biological clock can be changed or even reversed" – are counter to the current understanding of the field and imply that the clock itself is driving ageing rather than a 'biomarker' of the ageing process and the plethora of ageing-related changes it is capturing [1]. The clock itself is used to measure the impact of potential interventions [2].

We thank for the referee to point out this possible misunderstanding. It is widely accepted and demonstrated by various epigenome editing studies that DNA methylation is one of the most important factors that control gene expression, activation and splicing, hence many of the biological processes of the living systems. We agree that methylation is only one of the possible factors that control the ageing process and also that the 353 CpG-s in Horvath's clock give only a very small subset of them. We have reworded the abstract and explicitly stated that correlation is not equivalent to causation. We have also acknowledged that we demonstrate our approach only on a small set of CpG sites and to get biologically relevant control nodes, the analysis should be extended to all methylation sites.

In the revised version of the manuscript we mention the use of epigenetic clocks for the measurement of the impact of a thymus regeneration protocol as described in Ref.[2], whereas Ref.[1] was cited already in the original submission.

2. Furthermore, the statement that "adjustment of one leads to a cascade of changes at other sites" is not surprising if one understands what biological and connected epigenetic changes will be represented, as in this case of blood tissue derived DNA [3].

We agree, living things are complex interconnected systems. One of our goals with this paper was to emphasise this fact and to make the first step from the widely used linear models toward network model that may capture some of the complexities. We have reworded the cited sentence to avoid the false interpretation, and inserted a citation to Ref[3] from the referee report into the Introduction.

3. The statement in Abstract and elsewhere that 'we locate the most important CpGs' ignores the fact that they limit their analysis to only the 353 CpG from the total DNA methylome of 28 million CpGs to begin with. As Horvath has stated there is no evidence that the CpGs in the Horvath clock are especially functional over and above many other CpGs and reasonable clocks can be constructed from even a random selection of CpGs - there are abundant potential CpGs that can be exploited in clocks [3]. The statement "largest influence" and "which may also play a crucial role in the process of ageing" (Introduction, Line 94) again

implies these small fraction of 353 CpGs are uniquely special [4].

We have refined the mentioned statements, that refer specifically to the studied subset of CpGs in the revised version and put a caveat to the end of the Introduction to remind the reader that the analysis should be extended to get relevant results. (Ref.[4] from the referee report has been also incorporated into the manuscript, as described in the answer to Major point no. 9.)

4. Age-related change in DNA methylome is in fact widespread with up to 15 – 30% of all CpG sites in the genome associated with age-related changes and these are not all called 'clock CpGs' (Introduction line 18). Change can be random fashion due to epigenomic drift [5], directional, or show increased variability with age[6]. Also, the statement regarding the directionality of "clock CpGs that are hypermethylated" (Introduction line 35) is an oversimplification. Teschendorff et al. identified an enrichment in an early promoter-focused array for age-related CpGs that were hypermethylating in the Targets of Polycomb Target gene promoters, but genome-wide hypomethylation predominates. Both hypo- and hypermethylated loci contribute to the various published clocks.

We have rephrased the part of the text introducing the clock CpGs, now mentioning that age related CpGs are actually quite common, and that not all of them are called as clock CpGs. The revised version of the manuscript is now citing Refs[5,6] from the referee report. We also replaced 'hypermethilation' by 'age related change' in the sentence referring to the work by Teschendorff et al.

5. The statement in the Introduction that there are "connections between the CpGs themselves' (line 75) is as expected. Clearly all well-known ageing effects lead to co-ordinated changes across the entire DNA methylome – these include those driven by cell-type specific epigenomics where changes in cell proportion will led to variation (including the age-related myeloid skew [7], T cell exhaustion) [8], polycomb target hypermethylation [9], bivalent domain hypermethylation [10], etc. These known systemic effects will be seen as networks of age-related change.

We are especially grateful for this comment, providing extra support for the networked approach we use to study DNA methylation and ageing. This is now incorporated into the text (together with the references), however at a somewhat earlier point, where we first mention connections between the CpGs.

6. Distinct biological processes drive the observed age-related hypermethylation and hypomethylation. Furthermore, the baseline DNA methylation state is strongly driven by genetics being highly CpG density dependent [11].

We included this important point (together with the reference) in the revised version where we list the difficulties of constructing multi-tissue DNA methylation-based age estimators.

7. The statement (line 53) that "we cannot really point out any of these CpGs as being more important than others" is as completely expected in the way that the

elastic net regression Horvath clock was designed. CpGs were selected not for their individual strength but chosen for their power to work collectively to parsimoniously capture ageing over the lifecourse. In fact, this is clearly demonstrated by the fact the strongest and most robust individual CpG pan-tissue changes from the ELOVL2 locus [12,13] were not included in the clock. Additionally, an accurate clock has been devised using just 3 CpGs [14].

We agree that this is statement is somewhat evident, nevertheless we would like to keep it in the Introduction for helping non-expert readers in understanding the basis of our study. The sentence before this statement already mentioned that the correlation between age and the methylation of individual CpGs from Horvath's clock is weak; we have rephrased this sentence based on this comment, now citing Refs[12,13] from the referee report. Ref.[14] from the referee report was already cited in the original manuscript as Ref.[51] in the Discussion.

8. The discussion of "control properties" of CpGs is consistent with the Elastic Net picking those CpGs that work well together. Thus, the results regarding network identification and properties have ignored this and the limited CpGs this has been exacted from e.g. Results (line 112). Why were not all the 850,000 CpGs from the EPIC array analysed in the network analysis rather than just 353? Conclusion statements regarding how a "network approach can bring new insight into methylation-related studies, providing a very interesting direction for further research" (Line 389) are clearly limited when restricted to only these 353 CpGs and known biology not taken into account.

Analysing 850k (new EPIC array) or even 27k CpGs (older methylation array) is unfortunately not feasible computationally, due to the combinatorial explosion of the all-to-all nature of our analysis. This was the main reason why we have used only this limited set. In the updated version we call the readers' attention to this limitation.

The network we analysed can be viewed as a small sub-graph from the several orders of magnitude larger system of the whole methylome. A relevant related question is how do the interesting hierarchical and control properties we observed change when we scale up the network size? During the review process as a first step we have repeated our analysis on a network roughly 10 times larger obtained as follows. We took the 353 CpG dinucleotides in Horvath's clock one by one as a response variable, and carried Lasso regressions on the whole 450K CpG array appearing in the input data, where we marked the regressors (CpGs) obtaining a non-zero coefficient at least once. These marked CpGs along with the 353 CpGs in Horvath's clock defined an extended set of nodes, counting altogether 2036 CpGs. Among this larger set of nodes, the links were obtained based on LassoCV regression, following the network construction method described in the paper. We thresholded the links based on the absolute value of the regression coefficients to ensure that the average degree of the extended network becomes the same as in case of the original network studied in the paper.

The results of the hierarchy analysis on this extend network are shown in Fig.1.

As we can see, this network is again significantly more hierarchical compared to its random configuration model counterparts, similarly to the original network studied in the paper. Furthermore, the outcome of the control centrality analysis, shown in Fig.2., was also resembling to results we obtained for the network based solely on Horvath's clock.

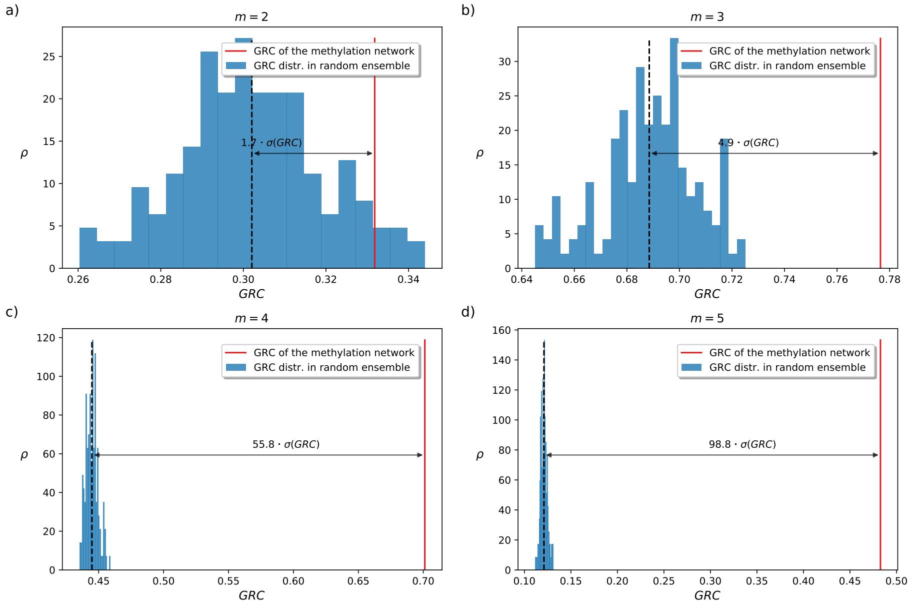

Fig. 1. Hierarchy of the extended methylation network. We show the GRC(m) measured for the network (red) together with probability density ρ(GRC) of the corresponding values in a link randomised ensemble of 150 networks (blue) at $m = 2$ (panel a), $m = 3$ (panel b), $m = 4$ (panel c), and $m = 5$ (panel d).

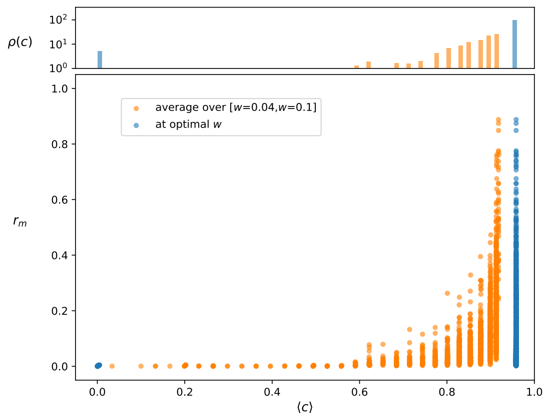

Fig. 2. Control centrality and reach in the extended methylation network. The main panel shows the reaching centrality r_m at $m = 3$ as a function of the relative control centrality c. Each symbol in the plot is corresponding to an individual CpG dinucleotide (node in the methylation network). In blue we show the results for the methylation network at the same link density as in the paper, whereas in case of the orange symbols $C(i)$ was averaged for the individual nodes over 30 different networks obtained by changing the link density around this optimal value.

According to these preliminary results, when we increase the size of the investigated methylation network by almost one order of magnitude, the hierarchical and control properties remain roughly the same. Naturally, this does not necessarily apply to the whole methylome, being still several orders of magnitude larger. Nevertheless, we believe that in the light of these results it is at least plausible that the properties of the network we investigated in the paper more or less reflect what we could expect for the entire network of CpG dinucleotides.

9. The authors need to explain and understand more precisely what the concept of 'biological age' and predicators of this represent [15]. The initial Horvath clock was devised as an attempt at a 'pan-tissue' clock (which it was highly successful in although caveats remain [16,17]). It is in fact a 'composite' clock [3] capturing both forensic and biological age but neither perfectly. The authors need to understand and integrate the current knowledge and issues regarding DNA methylation clocks - as discussed recently by the epigenomics community [4].

We revised the part in the Introduction mentioning the 'biological age' according to Refs.[3,15] in the referee report, which are now also cited in the manuscript. In addition, beside the success of Horvath's clock, we now mention the existence of related caveats together with citing Refs[16,17] from the referee report. Finally, key challenges and issues discussed in Ref.[4] of the referee report are also listed in the revised version (together with a citation to the paper).

10. The statements regarding "Modifying the predicted age by perturbing the methylation network' need to be put in the context that they are interpreting a 'biomarker' of biological ageing.

We have checked that we always refer to the adjustment of the "estimated" or "predicted" age and not true biological age. As indicated in the answers for other questions, we have put caveats concerning the interpretation both into the Introduction and Discussion.

11. Unclear what "more aligned with the 'natural direction of ageing'." (Line 283) means biologically?

The methylation values can be considered as coordinates of a multidimensional vector space. E.g. if we consider the 353 CpGs it will be a 353 dimensional space. Each patient's methylation measurement is a point in this space. Since methylation values are not random, the points do not cover the whole space, rather they are constrained to a (potentially curved) subspace. Projection techniques like the linear PCA or the recently popular non-linear t-SNE can reveal the most extended directions and are widely used to visualise the most important features of a high-dimensional data set. The principal directions can often be interpreted as biological features. For example the regression techniques used for age estimation identify such linear subspace. Changing few methylation values would move points according to the vector span by the linear combination of the corresponding axes, but the resulting position of the point may not necessarily stay on the "biologically allowed" subspace. As methylation values are part of an interacting network, change of one value cannot happen in isolation. In this part of the paper we describe this and show that by taking into account the cascading changes on our control network lead to changes that keep the points on the "biologically allowed" subspace in contrast to isolated (without following control cascades) changes that move points away from the subspace.

12. In the Discussion the statement 'Horvath's clock is showing non-trivial hierarchical and control properties' – how is this unexpected? Furthermore, how would that be different from a random selection of array-derived CpG probes?

In this study we represent the system of CpG dinucleotides as a network, and although we do not expect this to behave as e.g., an Erdős-Rényi random graph, still, the non-trivial nature of the interrelations can in principle be manifested in several different ways. E.g., a network can be different from a random graph in terms of its degree distribution, can display a community structure (that is absent in random graphs), may show assortativity or disassortativity, etc. In our view, it is not straightforward that a network ought to have a hierarchic structure (accompanied by interesting control properties) just because it represents biological data.

When considering a random baseline for comparison, we have to take into account that hierarchy measures are quite sensitive to the overall link density in networks. Based on that, we have chosen the configuration network ensemble to

serve as the baseline, where the random graphs correspond to uniformly drawn samples from all possible graphs with the same degree sequence as the original network, as mentioned in the Results section related to Fig.2. In this way we cancel out any possible uncertainty in the GRC coming from either a change in the overall link density or from a difference in the degree distribution.

Selecting random CpG probes is a very interesting idea, however, we would leave this to be the subject of further study, where also the size of the examined network might be increased (the first preliminary results of this analysis are described in the answer to Major comment no.8). Nevertheless, based on the results we have seen for the network of Horvath's clock, we expect both the entire network between all CpGs and randomly chosen sub-graphs from this to display hierarchical properties.

13. The statements regarding the functional implications of individual CpGs in the Discussion need to be more clearly caveated [8].

The description of the biological function of the genes was moved to the appendix (also because another referee found this part too long) and caveats were added.

14. In the Conclusion (line 374) the statement "substantially more hierarchical compared to a random Graph" does not take into consideration the biological nature of these data.

The concept of hierarchy in this work was introduced from a network theoretic point of view, e.g., the hierarchy measure we apply was used in social and technological networks as well in the literature. The random graph ensemble serving as a baseline preserves the degree distribution of the original network, thus, the most fundamental component of the network structure is not affected by the randomisation. In this light, the observation of a significantly higher GRC value in the original network compared to the random ensemble is already interesting from a pure network theoretic point of view. Nevertheless, we believe that this can be interesting for biologists as well, as it shows a non-trivial wiring between the CpG dinucleotides, where we can reach the majority of the network from nodes at the top of the hierarchy in just a few steps, whereas we cannot from bottom nodes.

Minor

1. English needs correcting throughout manuscript

The manuscript –according to the major comments and the points raised by the other referee– has been substantially rewritten.

2. Abstract – Grammar - "... biomarkers of ageing"

Corrected.

3. "specific CpG pairs" line 20 – CpG 'dinucleotides' is usually stated as more precise

Corrected.

4. Spelling line 33 - DNA methylation

Corrected.

5. Gene names are by convention written in italics – e.g. UCKL1 gene (line 314) etc.

Corrected.

1. Horvath, S. & Raj, K. DNA methylation-based biomarkers and the epigenetic clock theory of ageing. Nat Rev Genet 19, 371-384 (2018).

2. Fahy, G.M. et al. Reversal of epigenetic aging and immunosenescent trenűds in humans. Aging Cell 18, e13028 (2019).

3. Field, A.E. et al. DNA Methylation Clocks in Aging: Categories, Causes, and Consequences. Mol Cell 71, 882-895 (2018).

4. Bell, C.G. et al. DNA methylation Aging Clocks: Challenges & Recommendations. Genome Biology (2019).

5. Feil, R. & Fraga, M.F. Epigenetics and the environment: emerging patterns and implications. Nature reviews. Genetics 13, 97-109 (2011).

6. Slieker, R.C. et al. Age-related accrual of methylomic variability is linked to fundamental ageing mechanisms. Genome Biol 17, 191 (2016).

7. Rimmelé, P. et al. Aging-like Phenotype and Defective Lineage Specification in SIRT1-Deleted Hematopoietic Stem and Progenitor Cells. Stem Cell Reports 3, 44-59 (2014).

8. Lappalainen, T. & Greally, J.M. Associating cellular epigenetic models with human phenotypes. Nat Rev Genet 18, 441-451 (2017).

9. Teschendorff, A.E. et al. Age-dependent DNA methylation of genes that are suppressed in stem cells is a hallmark of cancer. Genome Res 20, 440-6 (2010).

10. Rakyan, V.K. et al. Human aging-associated DNA hypermethylation occurs preferentially at bivalent chromatin domains. Genome Res 20, 434-9 (2010).

11. Baubec, T. & Schübeler, D. Genomic patterns and context specific interpretation of DNA methylation. Current Opinion in Genetics & Development 25, 85-92 (2014).

12. Garagnani, P. et al. Methylation of ELOVL2 gene as a new epigenetic marker of age. Aging Cell 11, 1132-4 (2012).

13. Slieker, R.C., Relton, C.L., Gaunt, T.R., Slagboom, P.E. & Heijmans, B.T. Age-related DNA methylation changes are tissue-specific with ELOVL2 promoter methylation as exception. Epigenetics & Chromatin 11, 25 (2018).

14. Weidner, C.I. et al. Aging of blood can be tracked by DNA methylation changes at just three CpG sites. Genome Biol 15, R24 (2014).

15. Jylhävä, J., Pedersen, N.L. & Hägg, S. Biological Age Predictors. EBioMedicine 21, 29-36 (2017).

16. Horvath, S. et al. Epigenetic clock for skin and blood cells applied to Hutchinson Gilford Progeria Syndrome and ex vivo studies. Aging (Albany NY) 10, 1758-1775 (2018).

17. Zhang, Q. et al. Improved precision of epigenetic clock estimates across tissues and its implication for biological ageing. Genome Med 11, 54 (2019).

Response to Reviewer 2:

The study by Palla et al., entitled "Hierarchy and control age-related methylation networks", revealed that the age-related CpGs are interconnected, with dynamic methylation change on one CpG probably leading to a cascade of changes at the other sites. It provided a framework to explore the key methylation sites during ageing process, which might be applied to other biomarkers/biological processes. This study is interesting but remains too preliminary, as the authors only focused on the 353 Horvath's "clock CpGs". To better understand the issue raised in the study, a comprehensive analysis of CpGs involved in ageing and age-related phenotypes/diseases should be considered by collecting more methylation data. In this case, the manuscript needs to be revised thoroughly before considering for publication.

Thank you for your thorough review and comments. Analysing 850k (new EPIC array) or even 27k CpGs (older methylation array) is unfortunately not feasible computationally, due to the combinatorial explosion of the all-to-all nature of our analysis. This was the main reason why we have used only this limited set. In the Discussion of the updated version we call the readers' attention to this limitation. In the mean time, we intend to extend our research on larger methylation network in the future, our first preliminary results in this direction are described in the answer to Major concern no.8 by Reviewer 1. Based on that it seems plausible that the methylome may display interesting hierarchical and control properties when represented as a network on larger scales as well.

Major concerns: 1) The Introduction section is poorly summarized. Authors need simplify the content and clarify the background and purpose of the study.

The Introduction has been substantially rewritten due to the remarks by the other Referee; we hope that the background and the purpose of the study is more clear in the revised version.

2) Evidence supporting the leading roles of identified CpGs during ageing is insufficient. For example, the training model should be tested in multiple datasets. And, if possible, it will be great if some functional assays are performed.

We have refined our statements about the "top" CpGs of our analysis in ac-

cordance with the comments from the other Referee as well. Regarding the training of the model, we believe that the cross-validation technique involved in the regression analysis solves the problem this remark is pointing at. I.e., the data is sorted into 10 folds at random, and the Lasso regression is carried out on each fold separately. This is like having not a single, but instead 10 separate data sets (that are of course, necessarily smaller than the original). Finally, performing functional assays would be indeed the most reassuring, however, this is unfortunately beyond the capabilities of our group.

3) How shall we view the key CpGs' roles in ageing? It's hard to determine whether the methylation status is the result or the cause of ageing process. Authors should discuss this in Discussion section.

Thank you for calling our attention for this important issue. In the revised manuscript both in the Introduction and in the Discussion we put caveats concerning the interpretation of the role of the CpGs in Horvath's clock. Though limited to a very small set of methylation sites, our aim was to demonstrate a method, that steps beyond the usual linear regression approach and try to reveal the control structure, at least in silico.

4) Whether the training model can be used to scan the key CpGs that control various biological processes?

Thank you for this insight. Our method is general. Essentially it checks if the variation of the methylation at a CpG site can be estimated based on the values at other sites. The study in the paper was special in two senses. First, as discussed above we used a limited, potentially age-related subset of all CpGs in the genome. Second, the cohort was varied in the age of the patients. A larger or different subset of the CpG dinucleotides and a cohort with a variation in other biological processes would allow to reveal controller nodes for other processes. Since the article already relies on many hypotheses, we restrain from mentioning this possible extension in the paper.

5) Is there any correlation between a certain CpG's methylation status and its hierarchy level? (For example, sites located on higher levels may also have lower methylation values.)

This is a very interesting question, in Fig.3a we show the corresponding results. According to that, indeed, CpG dinucleotides on the higher hierarchy levels tend to have lower methylation values. In addition, also the variance of the methylation seems to be lower for nodes at the top of the hierarchy as indicated by Fig.3b.

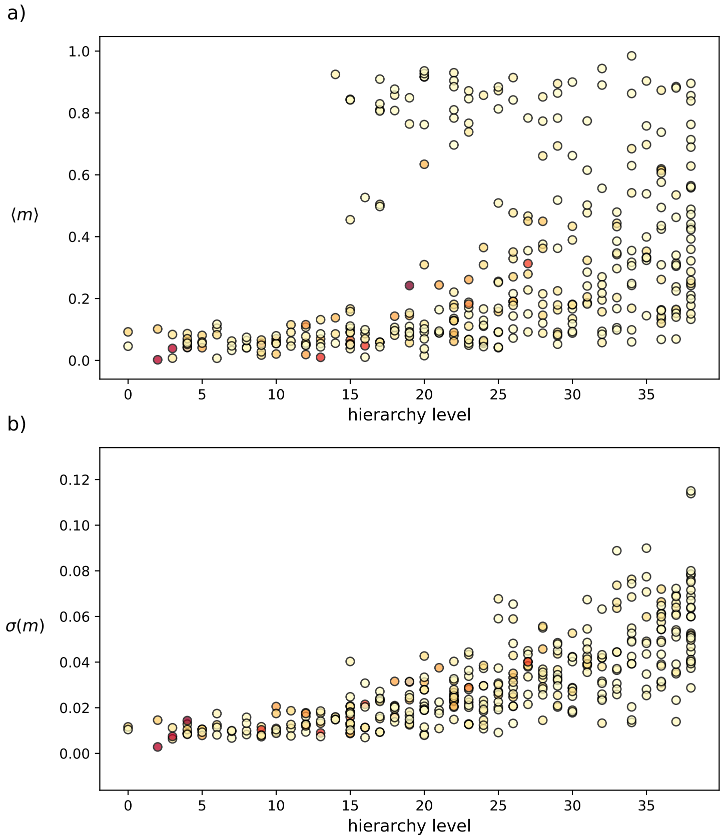

Fig. 3. Methylation and hierarchy levels a) The average methylation $\langle m \rangle$ over patients for the CpG dinucleotides in Horvath's clock as a function of their hierarchy level. (Node colours are taken from Fig.7. in the manuscript, indicating the the estimated age reduction value $|\Delta a|$ b) The standard deviation of the methylation.

6) The network is based on only 353 sites. When considering the CpGs across whole genome, the perturbation results may be different or even opposite. Authors may consider adding some data or results to demonstrate the robustness of the perturbation results. Generally, authors should, at least, provide evidence showing that the 353 "clock sites" are less affected by "non-clock sites".

We thank the suggestion, as mentioned in the answers to Referee 1, we have carried out a similar analysis on a larger network, containing the CpGs of Horvath's clock as a sort of "core". This network shows similar hierarchical and control properties to the original system we study in the manuscript, as shown in Figs.1-2. and is described in the answer to Major concern no.8 of Referee 1. We also calculated the age derivatives associated to the nodes in the extended network based on the same perturbation framework we detailed in the

manuscript. According to the results, the top 10 genes with the largest expected change in the estimated age are still corresponding to CpG dinculeotides appearing in Horvath's clock, 5 of which were in the top 10 also in the original study dealing with the smaller network. When we move to the top 20 instead, still there appears only 2 CpG dinucleotides that were not listed in Horvath's clock, however both of these belong to genes that also have another associated CpG dinucleotide in Horvath's clock with similarly high age derivative.

Nevertheless, we agree with the referee in that if the network is enlarged, the connections coming from "outside" are going to change the behaviour of the original "core" as well. In this light, when considering the whole human methylome, our results on the top CpG dinucleotides in Horvath's clock may have only limited relevance, and we now draw the attention of the reader to this in the Discussion of the revised text. In the mean time we believe that the observed hierarchical and control properties are likely to apply for larger methylation networks as well, and it is also very plausible that the correlation between the ability to induce change in the estimated age and the position in the hierarchy is general in this type of systems.

Minor concerns:

7) The Formula 4 doesn't render properly in the ms for reviewers.

Corrected.

8) The Discussion section seems too long, it talks too much on genes' functions. Authors may move and summarize these contents into the Results section.

We have created an Appendix, and moved the discussion on the possible biological roles of the top genes there, making the Discussion section itself much more concise.

Yours Sincerely,

Péter Pollner
