# Supplementary material for: Hierarchy and control of ageing-related methylation networks
Source: PLoS Comput Biol. 2021 Sep 17;17(9):e1009327. doi: 10.1371/journal.pcbi.1009327 (PMC8480875; doi:10.1371/journal.pcbi.1009327)
Supplement: Attachment — Submitted filename: pall_rereview_v1.pdf [file pcbi.1009327.s004.pdf]

Response to Reviewer 1:

We thank the Referee for the careful and detailed examination of the manuscript and the extremely valuable comments, which indeed have helped making our paper better. We are truly grateful for the 17 bibliographic references included in the report that we now also cited in the revised version of the paper. Our detailed answers to the points raised are the following

Palla et al. have produced a manuscript entitled “Hierarchy and control of ageing-related methylation networks.” In this paper they have extracted an interaction network from the CpGs employed in the Horvath clock. Unsurprisingly, this shows some hierarchical organisation is present. They then go on to discuss how modifying the clock will led to age `reversal'. Unfortunately, there are significant issues in the design and conclusions drawn from this study, due to imprecise understanding of the ageing biology of the epigenome, as well as the construction and interpretation of the Horvath clock. The researchers have performed a network analysis focused only the 353 CpGs from this specific clock, without acknowledging that these CpGs themselves are not uniquely special in regard to their functionality. The discussion of age-reversal gives these methylation sites a definitely active role in the ageing process which they do not possess.
My concerns are listed below.

Major
1. The statements in the Abstract that it is “plausible to assume that by proper adjustment of these switches age may be tuned" and that “biological clock can be changed or even reversed" are counter to the current understanding of the field and imply that the clock itself is driving ageing rather than a `biomarker' of the ageing process and the plethora of ageing-related changes it is capturing [1]. The clock itself is used to measure the impact of potential interventions [2].

We thank for the referee to point out this possible misunderstanding. It is widely accepted and demonstrated by various epigenome editing studies that DNA methylation is one of the most important factors that control gene expression, activation and splicing, hence many of the biological processes of the living systems. We agree that methylation is only one of the possible factors that control the ageing process and also that the 353 CpG-s in Horvath's clock give only a very small subset of them. We have reworded the abstract and explicitly stated that correlation is not equivalent to causation. We have also acknowledged that we demonstrate our approach only on a small set of CpG sites and to get biologically relevant control nodes, the analysis should be extended to all methylation sites. In the revised version of the manuscript we mention the use of epigenetic clocks for the measurement of the impact of a thymus regeneration protocol as described in Ref.[2], whereas Ref.[1] was cited already in the original submission.

- Good to acknowledge that causation differs from correlation. The point regarding ageing also specifically refers to the biological processes that are observed in blood as DNA methylation variation – as listed in regard to point 5 below.

2. Furthermore, the statement that “adjustment of one leads to a cascade of changes at other sites" is not surprising if one understands what biological and connected epigenetic changes will be represented, as in this case of blood tissue derived DNA [3].

We agree, living things are complex interconnected systems. One of our goals with this paper was to emphasise this fact and to make the first step from the widely used linear models toward network model that may capture some of the complexities. We have reworded the cited sentence to avoid the false interpretation, and inserted a citation to Ref[3] from the referee report into the Introduction.

- The point is not the complex system interconnectedness - but again what the multiple ageing-related changes in DNA methylation represent in blood (cell type changes etc.)

3. The statement in Abstract and elsewhere that "we locate the most important CpGs'" ignores the fact that they limit their analysis to only the 353 CpG from the total DNA methylome of 28 million CpGs to begin with. As Horvath has stated there is no evidence that the CpGs in the Horvath clock are especially functional over and above many other CpGs and reasonable clocks can be constructed from even a random selection of CpGs - there are abundant potential CpGs that can be exploited in clocks [3]. The statement "largest in influence" and "which may also play a crucial role in the process of ageing" (Introduction, Line 94) again implies these small fraction of 353 CpGs are uniquely special [4].
We have refined the mentioned statements, that refer specifically to the studied subset of CpGs in the revised version and put a caveat to the end of the Introduction to remind the reader that the analysis should be extended to get relevant results. (Ref.[4] from the referee report has been also incorporated into the manuscript, as described in the answer to Major point no. 9.)

- Good to acknowledge this caveat to this analysis.

4. Age-related change in DNA methylome is in fact widespread with up to 15-30% of all CpG sites in the genome associated with age-related changes and these are not all called `clock CpGs' (Introduction line 18). Change can be random fashion due to epigenomic drift [5], directional, or show increased variability with age[6]. Also, the statement regarding the directionality of "clock CpGs that are hypermethylated" (Introduction line 35) is an oversimpli_cation. Teschendorf et al. identified an enrichment in an early promoter-focused array for age-related CpGs that were hypermethylating in the Targets of Polycomb Target gene promoters, but genome-wide hypomethylation predominates. Both hypo- and hypermethylated loci contribute to the various published clocks.
We have rephrased the part of the text introducing the clock CpGs, now mentioning that age related CpGs are actually quite common, and that not all of them are called as clock CpGs. The revised version of the manuscript is now citing Refs[5,6] from the referee report. We also replaced 'hypermethylation' by 'age related change' in the sentence referring to the work by Teschendorf et al.

- The term 'clock CpGs' implies that these specific CpGs possess special properties. Therefore, this term only leads to confusion and would be better to remove – there are multiple CpGs in the DNA methylome that could be included, or not, into differently constructed DNA methylation clocks [Field et al].

5. The statement in the Introduction that there are "connections between the CpGs themselves" (line 75) is as expected. Clearly all well-known ageing effects lead to co-ordinated changes across the entire DNA methylome these include those driven by cell-type specific epigenomics where changes in cell proportion will led to variation (including the age-related

myeloid skew [7], T cell exhaustion) [8], polycomb target hypermethylation [9], bivalent domain hypermethylation [10], etc. These known systemic effects will be seen as networks of age-related change.

We are especially grateful for this comment, providing extra support for the networked approach we use to study DNA methylation and ageing. This is now incorporated into the text (together with the references), however at a somewhat earlier point, where we first mention connections between the CpGs.

- Good to now include this information.

6. Distinct biological processes drive the observed age-related hypermethylation and hypomethylation. Furthermore, the baseline DNA methylation state is strongly driven by genetics being highly CpG density dependent [11].

We included this important point (together with the reference) in the revised version where we list the diffculties of constructing multi-tissue DNA methylation-based age estimators.

- Good to now include this information.

7. The statement (line 53) that "we cannot really point out any of these CpGs as being more important than others" is as completely expected in the way that the elastic net regression Horvath clock was designed. CpGs were selected not for their individual strength but chosen for their power to work collectively to parsimoniously capture ageing over the lifecourse. In fact, this is clearly demonstrated by the fact the strongest and most robust individual CpG pan-tissue changes from the ELOVL2 locus [12,13] were not included in the clock. Additionally, an accurate clock has been devised using just 3 CpGs [14].

We agree that this is statement is somewhat evident, nevertheless we would like to keep it in the Introduction for helping non-expert readers in understanding the basis of our study. The sentence before this statement already mentioned that the correlation between age and the methylation of individual CpGs from Horvath's clock is weak; we have rephrased this sentence based on this comment, now citing Refs[12,13] from the referee report. Ref.[14] from the referee report was already cited in the original manuscript as Ref.[51] in the Discussion.

- However, the authors should include at this point why the specific methodology (elastic net) employed in the construction of the Horvath clock would contribute to this observation.

8. The discussion of "control properties" of CpGs is consistent with the Elastic Net picking those CpGs that work well together. Thus, the results regarding network identification and properties have ignored this and the limited CpGs this has been exacted from e.g. Results (line 112). Why were not all the 850,000 CpGs from the EPIC array analysed in the network analysis rather than just 353? Conclusion statements regarding how a "network approach can bring new insight into methylation-related studies, providing a very interesting direction for further research" (Line 389) are clearly limited when restricted to only these 353 CpGs and known biology not taken into account.

Analysing 850k (new EPIC array) or even 27k CpGs (older methylation array) is unfortunately not feasible computationally, due to the combinatorial explosion of the all-to-all nature of our analysis. This was the main reason why we have used only this limited set. In the updated version we call the readers' attention to this limitation. The network we analysed can be viewed as a small sub-graph from the several orders of magnitude larger system of the whole methylome. A relevant related question is how do the interesting hierarchical and control

properties we observed change when we scale up the network size? During the review process as a first step we have repeated our analysis on a network roughly 10 times larger obtained as follows. We took the 353 CpG dinucleotides in Horvath's clock one by one as a response variable, and carried Lasso regressions on the whole 450K CpG array appearing in the input data, where we marked the regressors (CpGs) obtaining a non-zero coefficient at least once. These marked CpGs along with the 353 CpGs in Horvath's clock defined an extended set of nodes, counting altogether 2036 CpGs. Among this larger set of nodes, the links were obtained based on LassoCV regression, following the network construction method described in the paper. We thresholded the links based on the absolute value of the regression coefficients to ensure that the average degree of the extended network becomes the same as in case of the original network studied in the paper. The results of the hierarchy analysis on this extend network are shown in Fig.1. As we can see, this network is again significantly more hierarchical compared to its random configuration model counterparts, similarly to the original network studied in the paper. Furthermore, the outcome of the control centrality analysis, shown in Fig.2., was also resembling to results we obtained for the network based solely on Horvath's clock.

- It would be good to demonstrate these in others clocks such as Hannan et al, GrimAge, SkinBlood etc to reinforce the biology.

9. The authors need to explain and understand more precisely what the concept of `biological age' and predicators of this represent [15]. The initial Horvath clock was devised as an attempt at a `pan-tissue' clock (which it was highly successful in although caveats remain [16,17]). It is in fact a `composite' clock [3] capturing both forensic and biological age but neither perfectly. The authors need to understand and integrate the current knowledge and issues regarding DNA methylation clocks - as discussed recently by the epigenomics community [4].
We revised the part in the Introduction mentioning the 'biological age' according to Refs.[3,15] in the referee report, which are now also cited in the manuscript. In addition, beside the success of Horvath's clock, we now mention the existence of related caveats together with citing Refs[16,17] from the referee report. Finally, key challenges and issues discussed in Ref.[4] of the referee report are also listed in the revised version (together with a citation to the paper).

- Good to acknowledge and expand on this important point.

10. The statements regarding “Modifying the predicted age by perturbing the methylation network” need to be put in the context that they are interpreting a `biomarker' of biological ageing.
We have checked that we always refer to the adjustment of the "estimated" or "predicted" age and not true biological age. As indicated in the answers for other questions, we have put caveats concerning the interpretation both into the Introduction and Discussion.

- Good to include these caveats.

11. Unclear what “more aligned with the 'natural direction of ageing'." (Line 283) means biologically?
The methylation values can be considered as coordinates of a multidimensional vector space. E.g. if we consider the 353 CpGs it will be a 353 dimensional space. Each patient's methylation measurement is a point in this space. Since methylation values are not random, the points do not cover the whole space, rather they are constrained to a (potentially curved) subspace.

Projection techniques like the linear PCA or the recently popular non-linear t-SNE can reveal the most extended directions and are widely used to visualise the most important features of a high-dimensional data set. The principal directions can often be interpreted as biological features. For example, the regression techniques used for age estimation identify such linear subspace. Changing few methylation values would move points according to the vector span by the linear combination of the corresponding axes, but the resulting position of the point may not necessarily stay on the "biologically allowed" subspace. As methylation values are part of an interacting network, change of one value cannot happen in isolation. In this part of the paper we describe this and show that by taking into account the cascading changes on our control network lead to changes that keep the points on the "biologically allowed" subspace in contrast to isolated (without following control cascades) changes that move points away from the subspace.

- The construction by elastic net will accentuation this interconnectedness.

12. In the Discussion the statement `Horvath's clock is showing non-trivial hierarchical and control properties' how is this unexpected? Furthermore, how would that be different from a random selection of array-derived CpG probes?

In this study we represent the system of CpG dinucleotides as a network, and although we do not expect this to behave as e.g., an Erdos-Renyi random graph, still, the non-trivial nature of the interrelations can in principle be manifested in several different ways. E.g., a network can be different from a random graph in terms of its degree distribution, can display a community structure (that is absent in random graphs), may show assortativity or disassortativity, etc. In our view, it is not straightforward that a network ought to have a hierarchic structure (accompanied by interesting control properties) just because it represents biological data. When considering a random baseline for comparison, we have to take into account that hierarchy measures are quite sensitive to the overall link density in networks. Based on that, we have chosen the configuration network ensemble to serve as the baseline, where the random graphs correspond to uniformly drawn samples from all possible graphs with the same degree sequence as the original network, as mentioned in the Results section related to Fig.2. In this way we cancel out any possible uncertainty in the GRC coming from either a change in the overall link density or from a difference in the degree distribution. Selecting random CpG probes is a very interesting idea, however, we would leave this to be the subject of further study, where also the size of the examined network might be increased (the first preliminary results of this analysis are described in the answer to Major comment no.8). Nevertheless, based on the results we have seen for the network of Horvath's clock, we expect both the entire network between all CpGs and randomly chosen sub-graphs from this to display hierarchical properties.

- A randomisation as well as analysis of the other clocks would help consolidate interpretation.

13. The statements regarding the functional implications of individual CpGs in the Discussion need to be more clearly caveated [8].

The description of the biological function of the genes was moved to the appendix (also because another referee found this part too long) and caveats were added.

- Good

14. In the Conclusion (line 374) the statement “substantially more hierarchical compared to a random Graph" does not take into consideration the biological nature of these data.

The concept of hierarchy in this work was introduced from a network theoretic point of view, e.g., the hierarchy measure we apply was used in social and technological networks as well in the literature. The random graph ensemble serving as a baseline preserves the degree distribution of the original network, thus, the most fundamental component of the network structure is not affected by the randomisation. In this light, the observation of a significantly higher GRC value in the original network compared to the random ensemble is already interesting from a pure network theoretic point of view. Nevertheless, we believe that this can be interesting for biologists as well, as it shows a non-trivial wiring between the CpG dinucleotides, where we can reach the majority of the network from nodes at the top of the hierarchy in just a few steps, whereas we cannot
from bottom nodes.

- The authors need to appreciate what these biological data represent to help explain why a hierarchical structure is observed.

Minor

- Good - all minor points have been corrected
