# Supplementary material for: Hierarchy and control of ageing-related methylation networks
Source: PLoS Comput Biol. 2021 Sep 17;17(9):e1009327. doi: 10.1371/journal.pcbi.1009327 (PMC8480875; doi:10.1371/journal.pcbi.1009327)
Supplement: Attachment — Submitted filename: Pollner et al_2ndRound.pdf [file pcbi.1009327.s006.pdf]

Pollner et al. have now provided a second round of responses to reviewers' queries, as below.
The authors have, commendably, performed extensive additional work, including further analysis of other epigenetic ageing clocks, random CpGs, and they have tempered statements regarding causation of these clock-related DNA methylation changes.

Response to Reviewer 1:

1. We hope that the amendments made in the previous and current version regarding points 1., 2., and 5., related to the relevant biological processes connected to methylation profile changes are now satisfactory.
2. We have made further changes to this part to avoid the possible interpretation that methylation level changes are the cause of ageing-related changes. In the current version we mention first the changes in cell proportion, including the age-related myeloid skew, T cell exhaustion, polycomb target hypermethylation, bivalent domain hypermethylation, which lead to coordinated modifications of the entire methylome, that in turn can be also interpreted as a network of age-related change in the methylation levels of the CpGs.
3. We thank the referee for accepting our response.
4. We have removed the term 'clock CpG' entirely from the paper.
5. We thank the referee for accepting our response.
6. We thank the referee for accepting our response.
7. In the new version we now explicitly mention when introducing Eq.(1) that Horvath's clock is based on elastic net regression. Furthermore, after the sentence referred in this point we inserted a short description of the parameter selection in the elastic net approach.
8. We have applied our framework to the Skin-Blood clock and Hannum's clock, receiving very similar results as already shown for Horvath's clock. The corresponding Figures have been placed into the Supporting Information (Sect.S2, Figs.S2-S7.) now accompanying the paper. The analysis for both of these further epigenetic clocks has shown that the methylation network composed of their CpGs is hierarchical, where the control centrality of the nodes is in positive correlation with the position of the nodes in the hierarchy. In addition, the chance to achieve a larger expected change in the estimated age when perturbing the methylation levels seemed to be higher for nodes close to the top of the hierarchy with large control centrality. These results are in very clear analogy with the results we discuss for Horvath's clock in the main text.
9. We thank the referee for accepting our response.
10. We thank the referee for accepting our response.
11. We have added a short reminder for the readers about the fact that CpGs in Horvath's clock were selected using elastic net regression.
12. Beside the analysis of the Skin-Blood clock and Hannum's clock we have also studied methylation networks composed of randomly chosen CpGs with a fixed size equal to that of Horvath's clock (353 nodes). The corresponding results are presented in the Supporting Information (Sect.S3, Figs.S8-S9.), indicating that these networks display quite similar properties compared to the previously studied networks representing epigenetic clocks. On the one hand, the hierarchy measure (the GRC) in their link randomised counterparts is on average lower compared to the GRC value of the original network structure encoding the inferred relations between the methylation levels. On the other hand, the control centrality of the nodes is in positive correlation with their position in the hierarchy. When comparing the GRC value obtained for Horvath's clock with the GRC distribution of the random methylation network we can observe that the hierarchy measure for Horvath's clock is above the

average at all studied m-parameters. However, its value is not an outlier, in the units of the standard deviation _ of the random distribution the difference is roughly between 1 σ and 2 σ, depending on m. Thus, the methylation network of Horvath's clock is resembling a methylation network with random CpGs where the hierarchy of the system is somewhat larger than the average, but not outstandingly large. By putting together the results obtained for networks representing epigenetic clocks and for the networks based on CpGs chosen uniformly at random from the 450k methylation array, we can conclude that basically any methylation network constructed according to our framework can be expected to display a hierarchical structure accompanied by control centrality values in positive correlation with the node position in the hierarchy. A remaining question of interest whether the hierarchy rankings obtained for small networks have an indicative value for the importance of the nodes we would observe in larger methylation networks where the size of set of CpGs taken into consideration is extended. Relating to that we have also examined mixed networks, where 10% of the CpGs were from the top of the hierarchy of Horvath's clock, and the rest of the nodes were chosen at random. The results (Fig.8. in the new version of the submission) show that hierarchy positions are conserved to a considerable extent across the different networks. This is promising for possible future research where the structure of larger parts from the methylome may be studied in small fragments analysed in a parallel fashion.
13. We thank the referee for accepting our response.
14. In regard to this point, the new version of the manuscript now mentions the relevant ageing related effects that are known to lead to coordinated methylation changes at this part of the Discussion.

All my concerns have been sufficiently answered
