# Supplementary material for: Hierarchy and control of ageing-related methylation networks
Source: PLoS Comput Biol. 2021 Sep 17;17(9):e1009327. doi: 10.1371/journal.pcbi.1009327 (PMC8480875; doi:10.1371/journal.pcbi.1009327)
Supplement: Attachment — Submitted filename: rebuttal_letter_3.pdf [file pcbi.1009327.s007.pdf]

Péter Pollner
Health Services Management Training Centre, Semmelweis University
H-1125, Kútvölgyi út 2,
Hungary

July 5, 2021

Editor
PLOS Computational Biology

Dear Editor of *PLOS Computational Biology*,

thank you very much for your e-mail message of the 20th of April 2021 regarding the 3rd round of reviews related to our manuscript PCOMPBIOL-D-20-00368 titled "Hierarchy and control of ageing-related methylation networks". Based on the replies given in the previous round, Referee 1 was satisfied, and stated that all of her/his concerns have been sufficiently answered. In the mean time, Referee 2 has raised an important criticism regarding the stability of our analysis over different methylation input data sets, pointing out that all of our previous studies were carried out on a single collection of methylation data.

In order to address this concern, we have applied our analysis framework to the further data set that was proposed in the Referee report. The obtained results are qualitatively very similar to what we have already observed in our previous studies: the methylation network is significantly more hierarchical compared to its link-randomised counterparts, the control centrality and the m-reach (determining the position in the hierarchy) are positively correlated and the best candidates for achieving a large change in the predicted age according to Horvath's clock by perturbing the methylation level turned out to be the CpGs with a large control centrality and positioned close to the top of the hierarchy. Furthermore, the hierarchy extracted from this new data set shows a reasonably high similarity with the hierarchy obtained for the original data set we used during the previous investigations, despite the seemingly large difference in the age distribution of the patient cohorts. In our view, these results confirm the robustness and stability of our analysis in general.

We have rewritten the manuscript by incorporating the details of the new results into the Supporting Information and providing a summary of the new findings in the manuscript. We express our gratitude to both Referees for their prominent work and their help in improving our paper, which now we hope is suitable for publication. Our detailed reply to the comment by Referee 2 in the last round (reproduced in italics) is given below.

Response to Reviewer 2:

In the new revision, the authors attempted to confirm the stability of the hi-

erarchy of ageing-related networks by considering the CpG sites in Skin-blood clock and Hannum's clock. However, these analyses are still performed by using only one Human Methylation 450K dataset (GSE40279) even the reviewer has already pointed out last time. This strategy does not support the view that the finding for the CpG sites of concern is general. As the authors explained – "Analysing 850k (new EPIC array) or even 27k CpGs (older methylation array) is unfortunately not feasible computationally, due to the combinatorial explosion of the all-to-all nature of our analysis" or "Scaling up the network size in our analysis to the level of the whole 450k array would take more than 150 years; thus, it is truly not feasible", but it means that the analysis of CpG sites in 'Epigenetic Clock' (e.g., Horvath's clock) in another independent methylation dataset (e.g., GSE55763 that contains over 2600 samples) is theoretically possible and worthy, even perhaps I underestimated the difficulty. Overall, I have to say the current revision is still inconclusive.

We agree that all of our previous studies were based solely on the GSE40279 dataset, and an important direction for generalising the results is to involve further datasets, such as the GSE55763 dataset. During the revision we addressed this issue, and have examined the methylation network that can be extracted based on the GSE55763 dataset according to our analysis framework.

However, before going into the details, we would like to point out a very important difference between this dataset and the previously used GSE40279 dataset. According to Fig.S16 in the revised version of the Supporting Information, the age distribution of the patients in the GSE55763 dataset is concentrated on a significantly shorter interval compared to the GSE40279 dataset, and the two distributions show a rather different shape even within this shorter interval. In more details, the age of patients can only fall between 23 and 75 years for the GSE55763 dataset, whereas in contrast it is spanning between 19 and 101 years for the GSE40279 dataset, where also a relatively large fraction of the patients is above 75 years old.

Since the methylation profile is changing with ageing (making the development of epigeneitc clocks possible in the first place), the above mentioned differences should be kept in mind when comparing the results of our analysis pipeline for the two datasets. Although the inclusion of this extra dataset is indeed instructive, and provides important information regarding the generality of our results, based on the large differences in the age distributions, a perfect match between e.g., the methylation hierarchies cannot be expected.

Our results related to the GSE55763 dataset are detailed in Sect.S4. of the revised version of the Supporting Information. As already mentioned, the qualitative behaviour of the obtained methylation network is very similar to the previous results obtained for the GSE40279 dataset. According to Fig.S10. the hierarchy measured according to the GRC is outstandingly high compared to the GRC distribution in the configuration model ensemble equivalent of the network. The control centrality and the m-reach show positive correlation as

indicated by Fig.S11., and finally, when plotting the magnitude of the expected change in the predicted age under perturbation of the methylation level as a function of both the m-reach and the control centrality as in Fig.S12., we can observe an overall increasing tendency in the point cloud.

We also compared the hierarchy between the CpGs based on the GSE55763 dataset to the earlier hierarchy obtained for the GSE40279 dataset in Figs.S13-S15. We found that despite the mentioned significant differences between the age distributions of the patient cohorts, the two alternative hierarchies show a reasonably high similarity. E.g., the Pearson correlation between the m-reach (determining the position in the hierarchy) calculated in the two networks is $C = 0.75$, and the top 10% of the nodes according to one hierarchy is concentrated close to the top in the other hierarchy as well. In our opinion, these findings indicate that our analysis framework is stable, and the results obtained over multiple input datasets show a relatively high consistency within the inherent bounds set by the deviations between the two patient cohorts.

Naturally, we also inserted a summary of these new findings into the main paper in Sect. Extension of the analysis to further methylation networks, and updated the Discussion as well. Meanwhile, since the qualitative behaviour of this new methylation network is very similar to the previous results seen for the GSE40279 dataset, we would like to keep the corresponding figures and the detailed description in the Supporting Information, in order to allow the main text to remain focused. We hope that in the light of the above discussed new results, the revised version of the paper is now suitable for publication.

Yours Sincerely,

Péter Pollner
